# Supplementary material for: Bik reduces hyperplastic cells by increasing Bak and activating DAPk1 to juxtapose ER and mitochondria
Source: Nat Commun. 2017 Oct 6;8:803. doi: 10.1038/s41467-017-00975-w (PMC5630627; doi:10.1038/s41467-017-00975-w)
Supplement: Supplementary file 1 — Supplementary Information [file 41467_2017_975_MOESM1_ESM.pdf]

Supplementary Figure 1

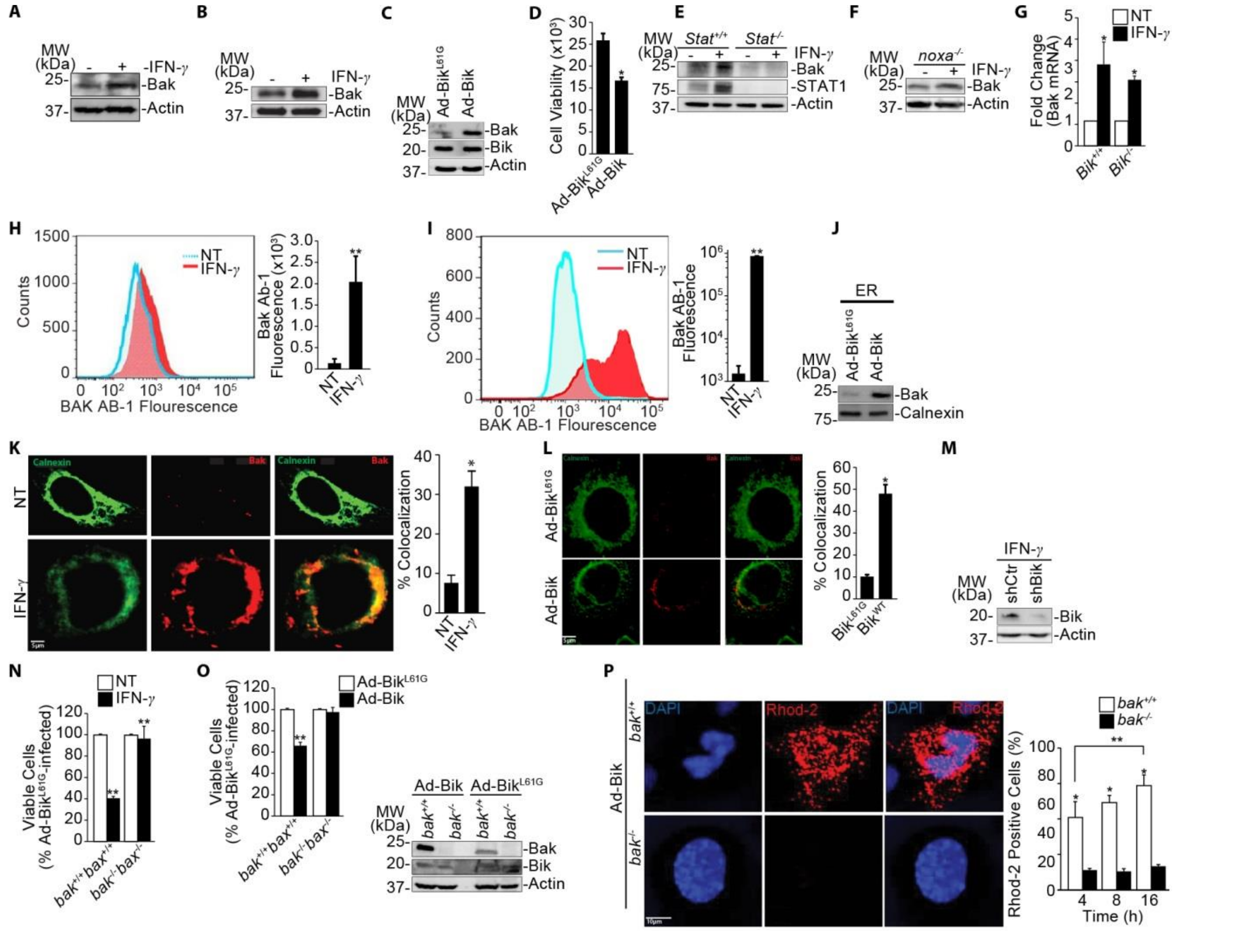

Supplementary Figure 1: (A) Primary human airway epithelial cells or **(B)** MAECs were treated with 50 ng/ml IFN-γ or medium alone and protein lysates were analyzed for changes in the expression of Bak by Western blot. (**C, D**) Primary colon epithelial cells were isolated from C57BL/6 mice. Cells were plated on 6-well plates and infected with 100 MOI Ad-Bik^{L61G} or Ad-Bik. **(C)** Protein lysates analyzed for Bak expression levels by Western blotting. **(D)** Cells were quantified 24 h later. MAECs isolated from **(E)** *stat*$^{+/+}$ or *stat*$^{-/-}$ or **(F)** *noxa*$^{+/+}$ or *noxa*$^{-/-}$ mice treated with 50 ng/ml murine recombinant IFN-γ or left untreated as controls and protein lysates were analyzed for Bak expression 24 h after treatment by Western blot. **(G)** *bik*$^{+/+}$ and *bik*$^{-/-}$ MAECs were treated with 50 ng/ml IFN-γ or medium alone and Bak mRNA levels were analyzed 24 h later by qRT-PCR. HAECs treated with IFN-γ or medium alone as controls in the presence of 20 µM pan-caspase inhibitor Q-VD-OPh for 24 h **(H)** or 72 **(I)** were analyzed for active Bak (Ab-1)-specific fluorescence by FACS. **(J)** HAECs infected with 100 MOI Ad-Bik or Ad-Bik^{L61G} for 24 h**,** the ER fraction of the protein lysates were analyzed for active Bak by Western blot. **(K)** Representative photomicrographs of HAECs treated with or without 50 ng/ml IFN-γ for 48 h in the presence of 20 µM pan-caspase inhibitor Q-VD-OPh, fixed with paraformaldehyde and immunostained for activated Bak (Ab1) and calnexin. **(L)** Representative photomicrographs of HAECs infected with 100 MOI Ad-Bik or Ad-Bik^{L61G} for 24

h, fixed with paraformaldehyde and immunostained for activated Bak (Ab1) and calnexin. **(M)** HAECs stably expressing shCtr or shBik were treated with 50 ng/ml IFN-γ for 48 h. Protein lysates were analyzed for knockdown of Bik by Western blot. *bak*$^{+/+}$/*bax*$^{+/+}$ or *bak*$^{-/-}$*bax*$^{-/-}$ HCT116 cells were treated with medium alone or 50 ng/ml human recombinant IFN-γ for 48 h **(N)** or infected with 100 MOI Ad-Bik^{L61G} or Ad-Bik for 24 h **(O)**. Cells were quantified and protein lysates were analyzed by Western blot. **(P)** MAECs from *bak*$^{+/+}$ or *bak*$^{-/-}$ were infected with 100 MOI Ad-Bik for 4, 8, or 16 h and stained with (Ca^{2+})m indicator, Rhod-2 (Red), counterstained for nuclei with DAPI (blue) and the percentage of cells with Rhod-2 positivity were quantified. Percent localization for each experiment in K and L were calculated by counting 200 cells. Scale bar, 5 μm. Differences between two groups were assessed for significance by Student's t test. ANOVA was used to perform pair-wise comparison of data from more than two groups followed by Fisher least significant difference test. Error bars indicate ± SEM, *=P<0.05; **=P<0.01.

Supplementary Figure 2

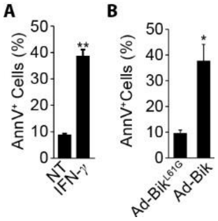

Supplementary Figure 2: (A) HAECs were treated with 50 ng/ml human recombinant IFN-γ for 72 h and analyzed for annexin V positivity by FACS. **(B)** HAECs were infected with Ad-Bik or Ad-Bik^{L61G} and 24 h later analyzed for Annexin V positivity by FACs. Differences between two groups were assessed for significance by Student's t test. Error bars indicate ± SEM, *=*P*<0.05; **=*P*<0.01.

Supplementary Figure 3

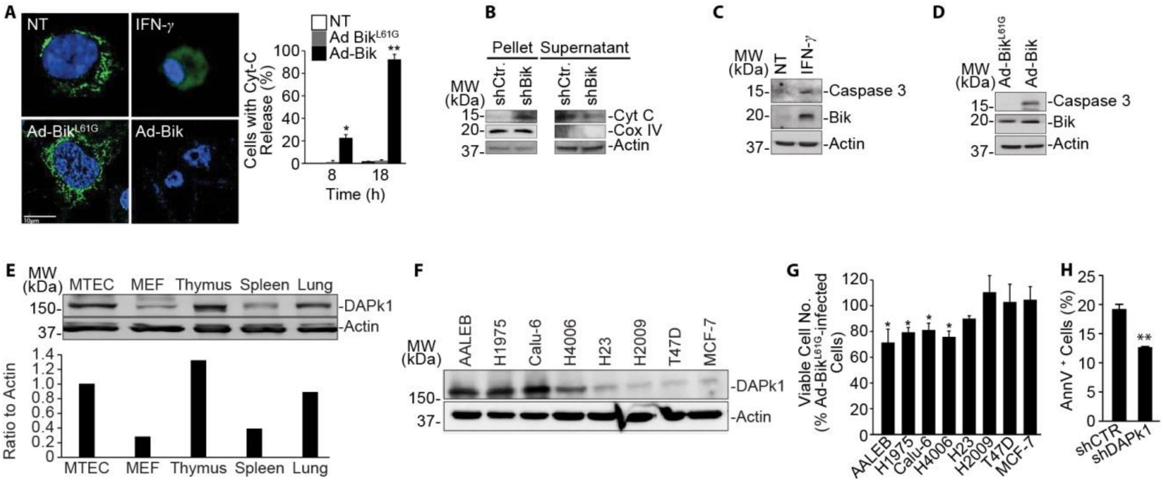

Supplementary Figure 3: (A) H1299 cells stably expressing Cyt C were infected with 100 MOI Ad-Bik or Ad-Bik^{L61G} or treated with 50 ng/ml IFN-γ or medium alone. Cells were analyzed for Cyt C release at 8 or 18 h post treatment by fluorescent microscopy. **(B)** HAECs stably expressing shCtr for Bik or shBik were infected with 100 MOI of Ad-Bik for 18 h. Protein lysates were fractionated into mitochondrial pellets and cytosolic supernatants and subjected to Western blotting. Cell lysates prepared from HAECs treated with 50 ng/ml human IFN-γ or left NT **(C)** or infected with 100 MOI Ad-Bik or Ad-Bik^{L61G} for 24 h **(D)** were probed for cleaved caspase 3 by Western blotting. **(E)** Protein lysates from MTEC, MEF, Thymus, spleen and lung were analyzed for the expression of DAPk1 by Western blot. **(F)** Protein lysates from HAECs, H1975, Calu-6, H4006, H23, H2009, HT47D and MCF-7 cell lines were compared for the expression level of DAPk1 by Western blot. **(G)** Cells were infected with 100 MOI of Ad-Bik or Ad-Bik^{L61G} and analyzed for cell viability 24 h later. For all experiments n=3 independent experiments. **(H)** HAECs stably expressing shCtr or shDAPk were analyzed for Annexin V-positivity by FACs 24 h after infection with100 MOI Ad-Bik. Differences between two groups were assessed for significance by Student's t test. ANOVA was used to perform pair-wise comparison of data from more than two groups followed by Fisher least significant difference test. Error bars indicate ± SEM, n=5; *=p<0.05; **= p<0.01.

Supplementary Figure 4

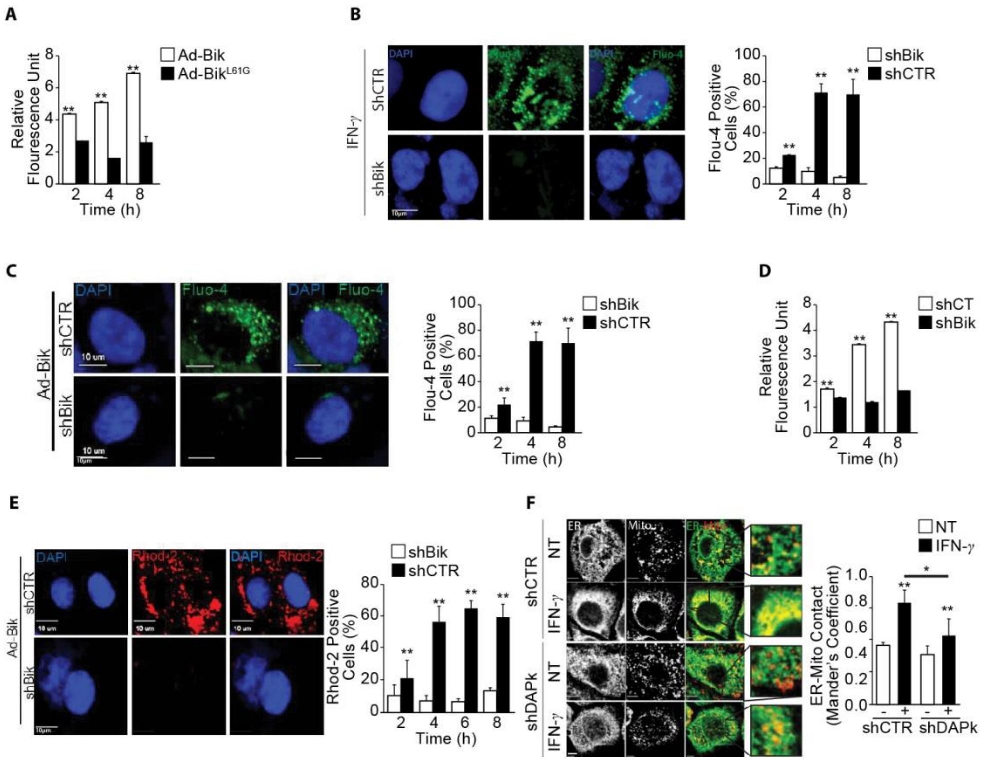

Supplementary Figure 4: (**A**) HAECs were infected with Ad-Bik or Ad-Bik^{L61G} for 6, 8, or 16 h. Cells were stained with Fluo-4 and ER-calcium release was analyzed using fluorimeter. **(B)** HAECs stably expressing shCTR or shBik were treated with 50ng/ml IFN-γ for the indicated time point and stained with Ca^{2+}-flux indicator, Fluo-4 (green) and counterstained for nuclei with DAPI (blue) and the percentage of cells with Fluo-4-positivity were quantified. **(C)** HAECs stably expressing shCtr or shBik were infected with Ad-Bik for the indicated time point and stained with Fluo-4 (green) and counterstained for nuclei with DAPI (blue) and the percentage of cells with Fluo-4-positivity were quantified. **(D)** HAECs stably expressing shBik or shCtr were infected with Ad-Bik for 6, 8, or 12 h and ER-calcium releases were analyzed using fluorimeter. **(E)** HAECs stably expressing shCtr or shBik were infected with Ad-Bik for 2, 4, and 8 h, stained with and $[Ca^{2+}]_m$ indicator Rhod-2 (red), counterstained for nuclei with DAPI (blue), and the percentage of cells with Rhod-2-positivity were quantified. Graphs show mean ± SEM of quantified cells with released Ca^{2+} (Rhod-2 positive cells (n=3, with >200 cells analyzed per condition. **(F)** Representative micrographs of shCtr- and shDAPk1-expressing HAECs treated with 50 ng/ml recombinant human IFN-γ or medium alone (NT) as control**.** Cells were stained with anti-CoxIV for mitochondria (green), with anti-calnexin for ER (red), and with DAPI for nuclei staining (blue). Confocal images of 3D reconstructions of ER and mitochondria were acquired. Scale bars, 5 µm. ER-mitochondria contacts were quantified by Manders' coefficient, means and SEM (n=5, at least 40 cells per experiment) of morphometric data. (n=5, with >200 cells analyzed per condition). For all experiments n=3 independent experiments. Differences between two groups were assessed for significance by Student's t test. ANOVA was used to

perform pair-wise comparison of data from more than two groups followed by Fisher least significant difference test. Error bars indicate ± SEM, *=p<0.05; **= p<0.01.

Supplementary Figure 5

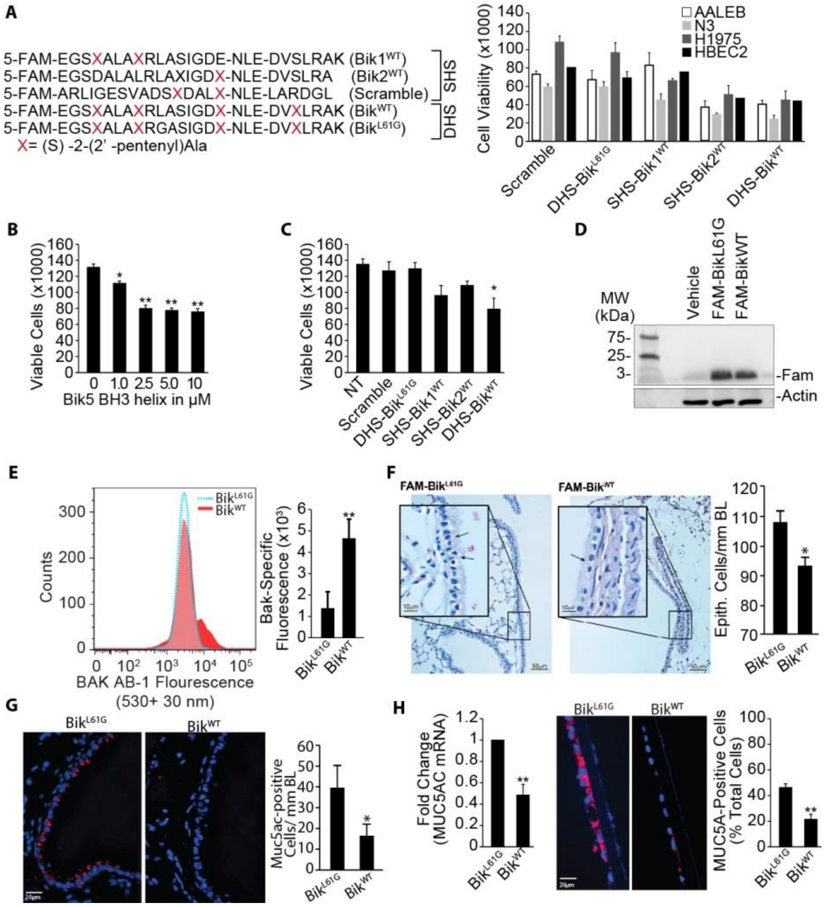

Supplementary Figure 5: (A) Sequence composition of FAM-Bik BH3 and its L61G mutant peptides screened for their effect in causing cell death in different cell lines. “X” denotes the position of the non natural amino acid with (S)-2-(2’-pentenyl) Ala residues within each peptide. NHBEs (AALEB, N3, HBEC2) and EGFR mutant cancer cell line (H1975) were plated in 24-well plates and treated with 5 µM scramble, DHS-Bik^{L61G}, SHS-Bik2 or DHS-Bik^{WT} peptides. Cells were harvested and quantified 72 h later. **(B)** AALEB cells were seeded on 6-well plates and treated with 0, 1.0, 2.5, 5.0, or 10 µM DHS-Bik^{WT} BH3 helix and quantified 72 hours later. There was no significant difference in sensitivity of the cells between 2.5, 5.0, and 10 µM treatments. **(C)** Primary normal human bronchial epithelial cells (NHBEs) from five donors were plated in 24-well plates and treated with 5 µM of single (SHS-Bik1^{WT}, SHS-Bik2 ^{WT}), double (DHS-Bik^{WT}, DHS-Bik^{L61G}), or scramble hydrocarbon stapled Bik BH3 helixes. Cells were harvested and quantified 72 hours later. DHS-Bik^{WT} was found to be the most effective in reducing cell number. **(D)** Cultured HAECs were treated with vehicle, FAM-Bik^{L61G} or FAM-Bik^{WT} BH3 peptides (1 µM) for 2 hours, followed by centrifugation, trypsinization, washing, lysate preparation, electrophoresis, and fluorescence detection. **(E)** HAECs treated with 5 µM of FAM-DHS-Bik^{WT} or FAM-DHS-Bik^{L61G} peptides for 3 h were analyzed for active Bak (Ab1-specific) fluorescence by FACS. **(F, G)** C57BL/6 mice were exposed to 250 mg/m^3 CS 6 h/d, 5 d/wk for 3 weeks and instilled with 5 uM of FAM-DHS-Bik^{WT} or FAM-DHS-Bik^{L61G} peptides in 50ul of PBS on 2

consecutive days and lungs harvested the following day. The number of epithelial cells **(F)** Muc5ac-positive cells per mmBL and **(G)** were significantly reduced in the airways of mice instilled with FAM-DHS-Bik^{WT} compared with those instilled with FAM-DHS-Bik^{L61G} or PBS. **(H)** Differentiated HAECs were treated with 4000 ng/ml CSE for 48 h followed by treatments with 5 uM of FAM-Bik^{WT} or FAM-DHS-Bik^{L61G} peptides for 48 h before harvest. FAM-DHS-Bik^{WT} peptides reduce MUC5AC mRNA levels and percentage of MUC5AC-positive cells in CS-treated cells as analyzed by qRT-PCR and immunostaining, respectively. FAM-DHS-Bik^{WT} reduced MUC5AC positivity significantly compared to FAM-DHS-Bik^{L61G}. (n = 5 individuals/group, done in triplicate). Differences between two groups were assessed for significance by Student's t test. ANOVA was used to perform pair-wise comparison of data from more than two groups followed by Fisher least significant difference test. Error bars indicate ± SEM, Graphs and error bars show means±SEM, *=p<0.05; **= p<0.01.

Supplementary Figure 6. Uncropped images related to Figure 1

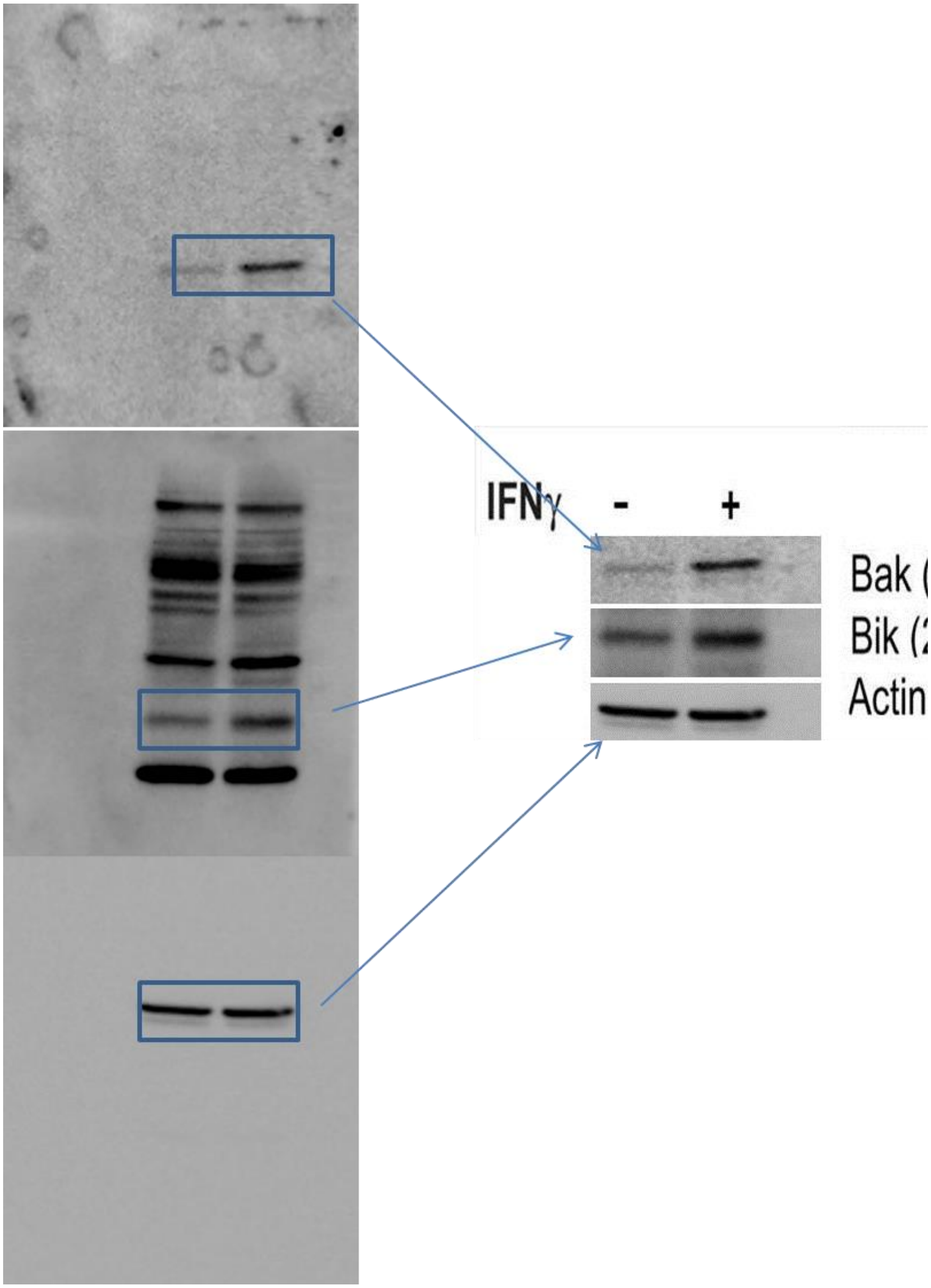

Figure 1A

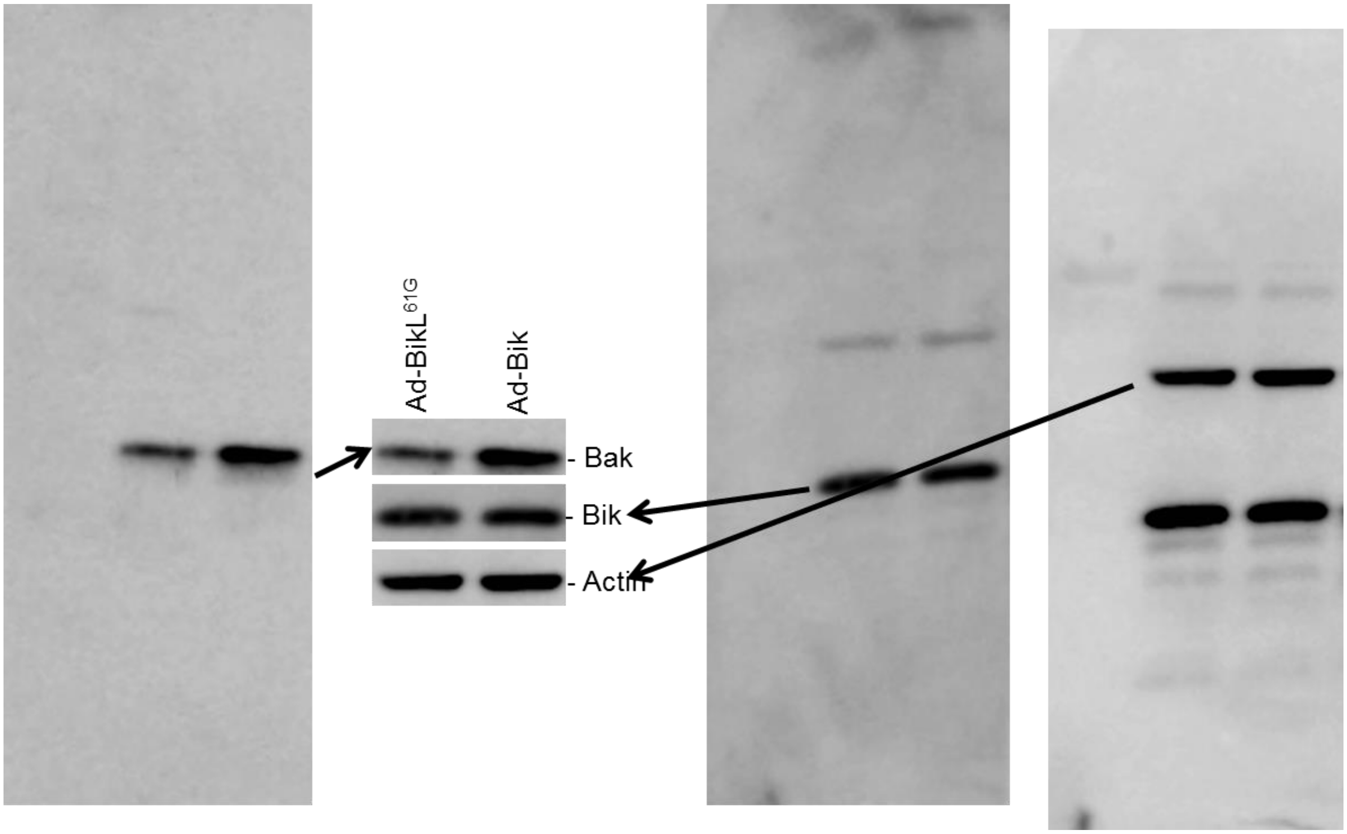

Figure 1B

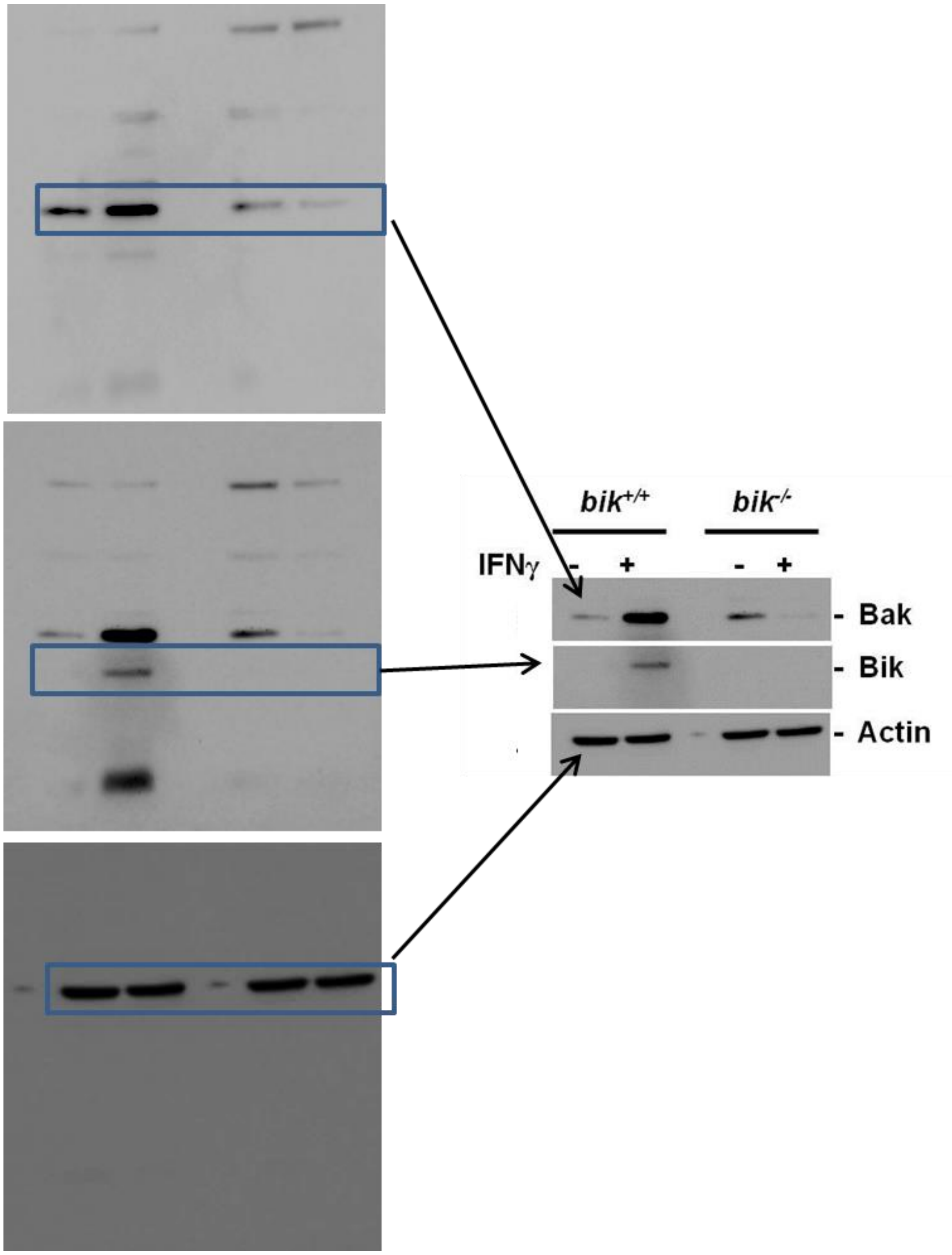

Figure 1C

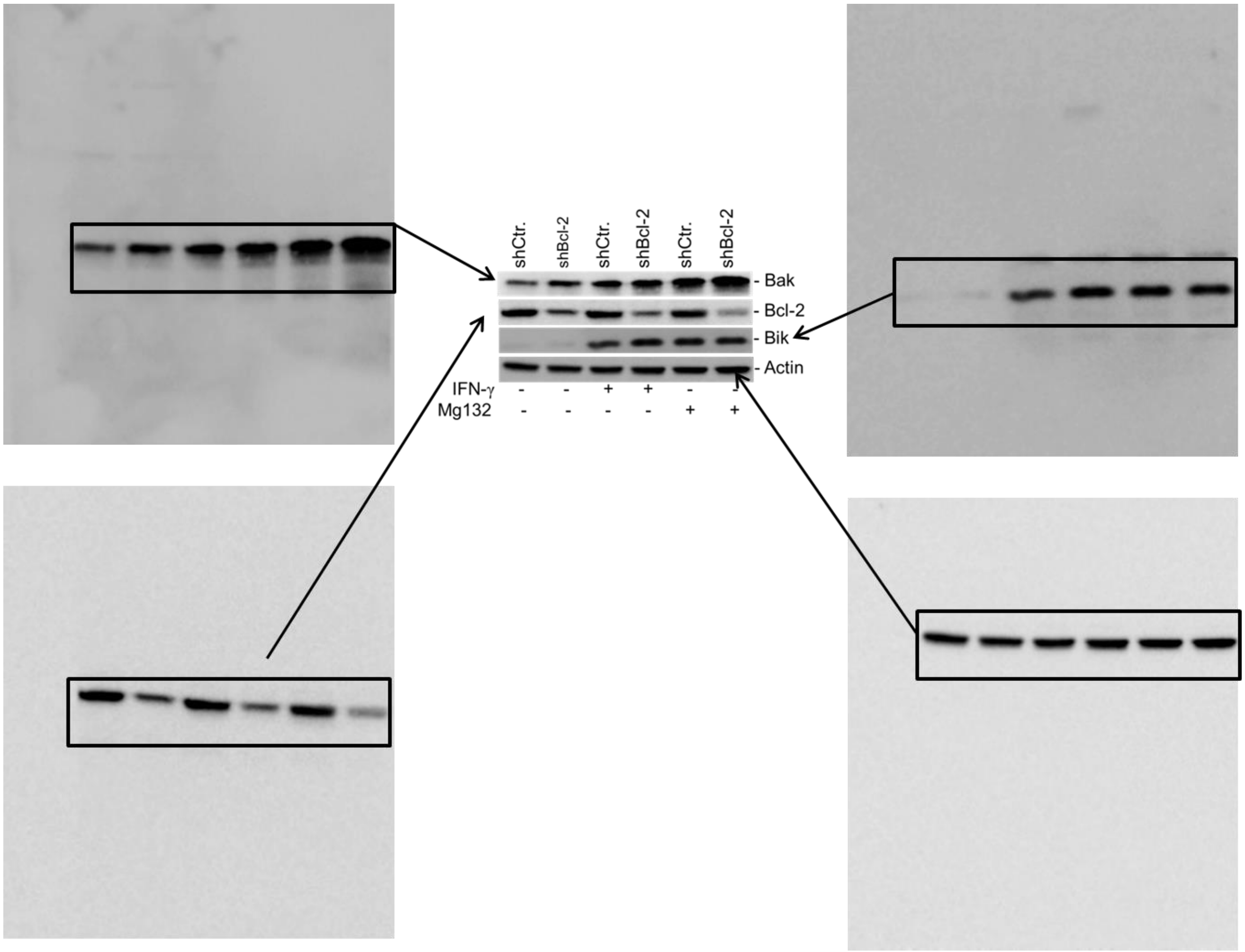
shCtr.
shBcl-2
shCtr.
shBcl-2
shCtr.
shBcl-2
- Bak
- Bcl-2
- Bik
- Actin
IFN-γ - - + + - -
Mg132 - - - - + +

Figure 1D

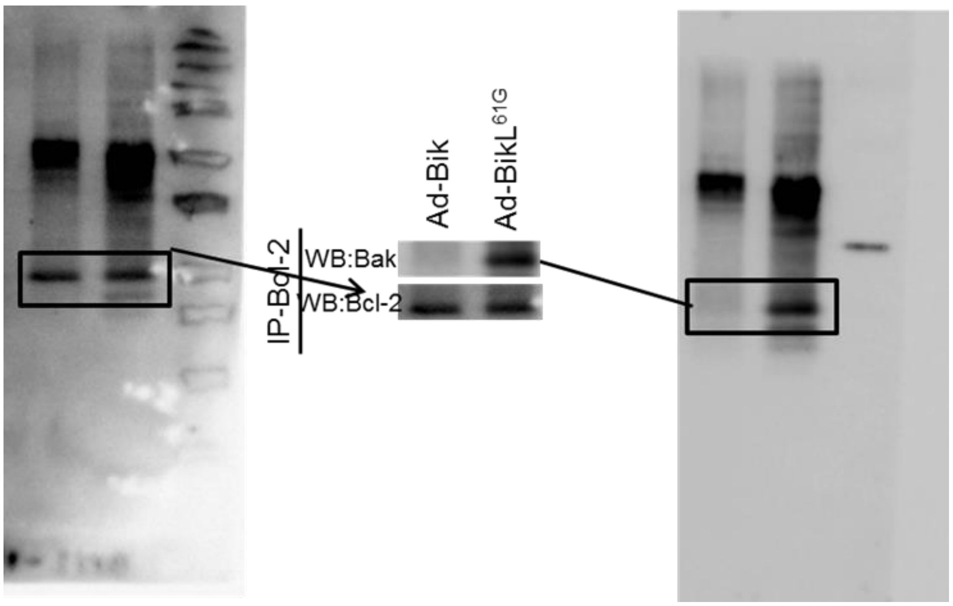
Ad-Bik
Ad-BikL61G
IP-Bcl-2
WB:Bak
WB:Bcl-2

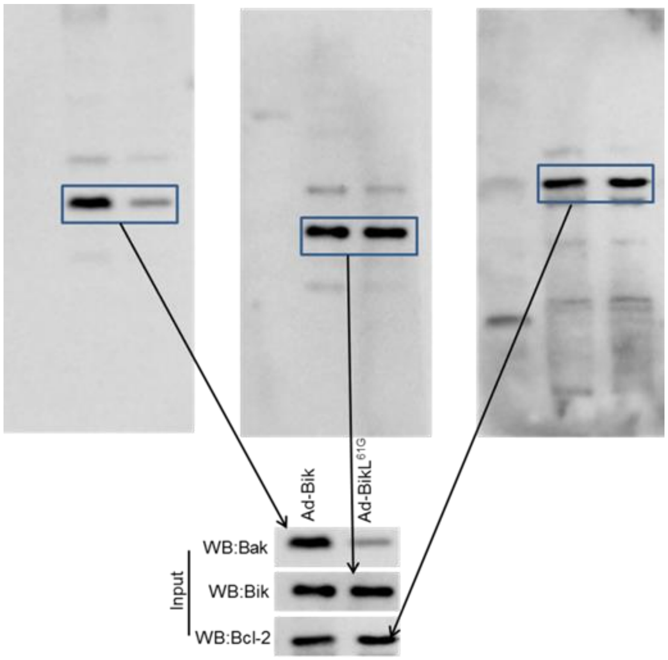
Ad-Bik
Ad-BikL61G
Input
WB:Bak
WB:Bik
WB:Bcl-2

Figure 1E

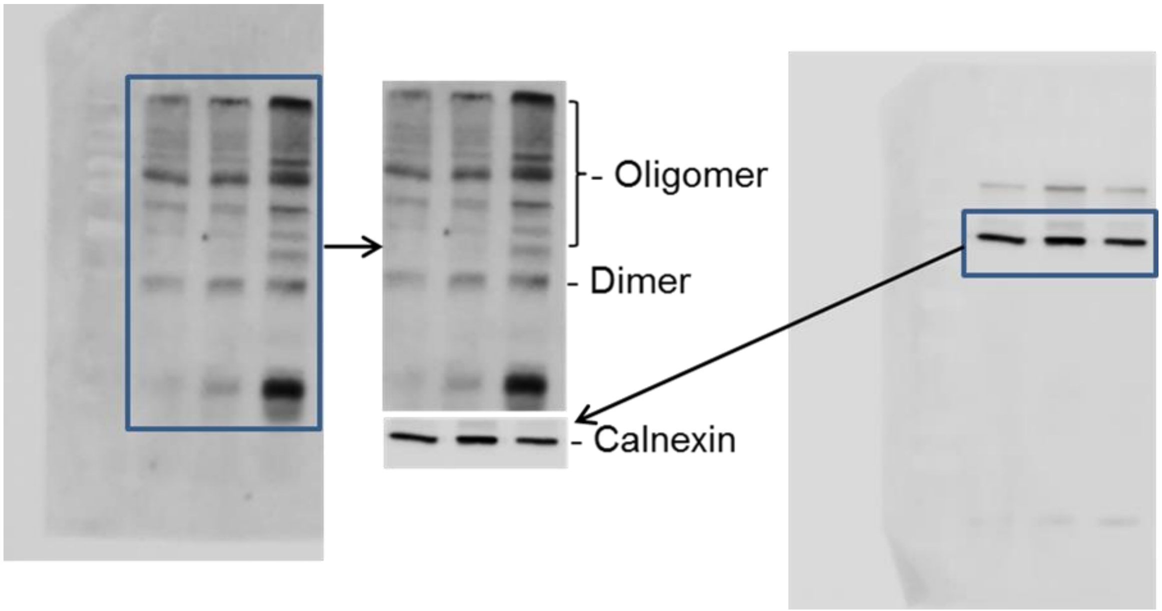

Figure 1G

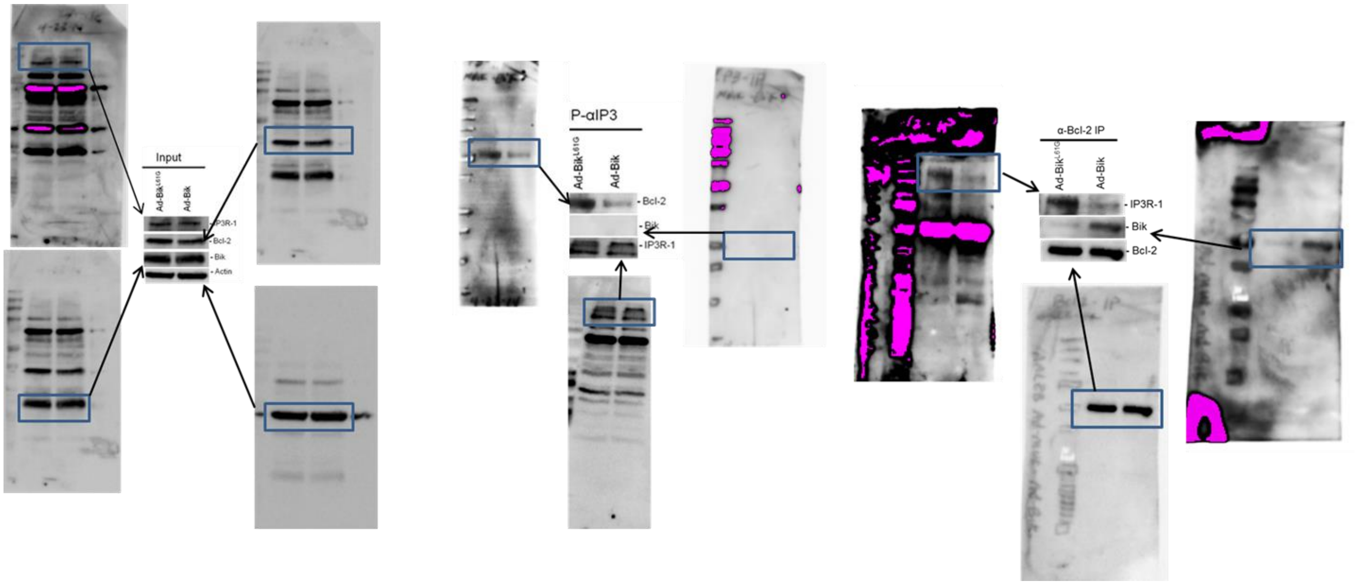
Input
Ad-Bik^{L61G}
Ad-Bik
IP3R-1
Bcl-2
Bik
Actin
P-αIP3
Bcl-2
Bik
IP3R-1
α-Bcl-2 IP
IP3R-1
Bik
Bcl-2

Figure 1K

Supplementary Figure 7. Uncropped images related to Figure 2

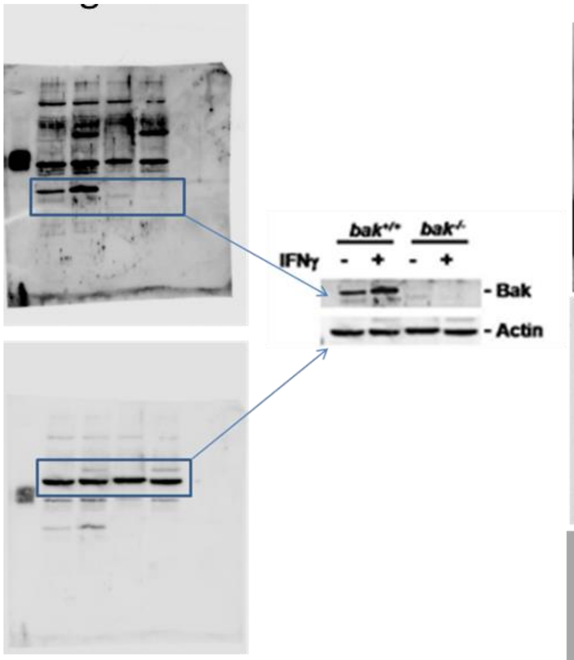

Figure 2A

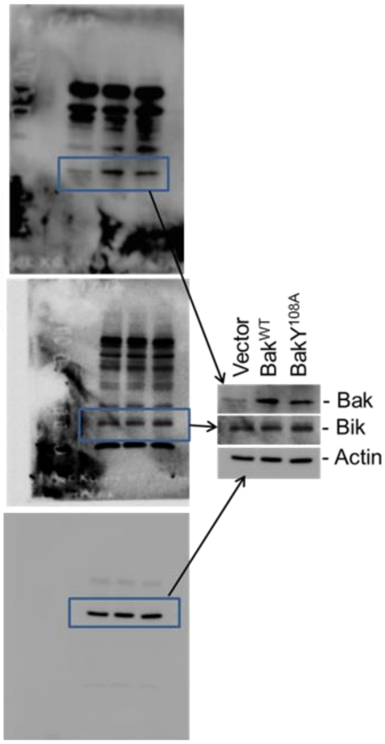

Figure 2C

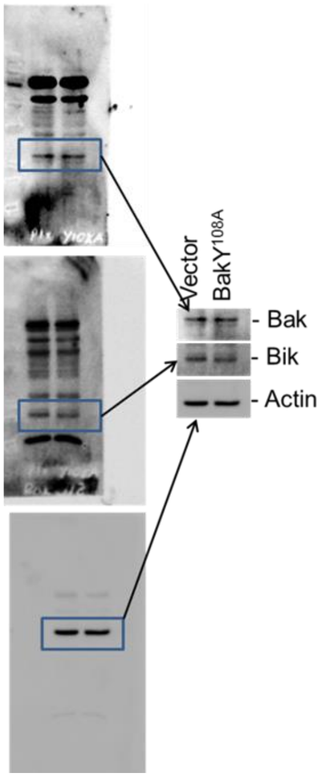

Figure 2D

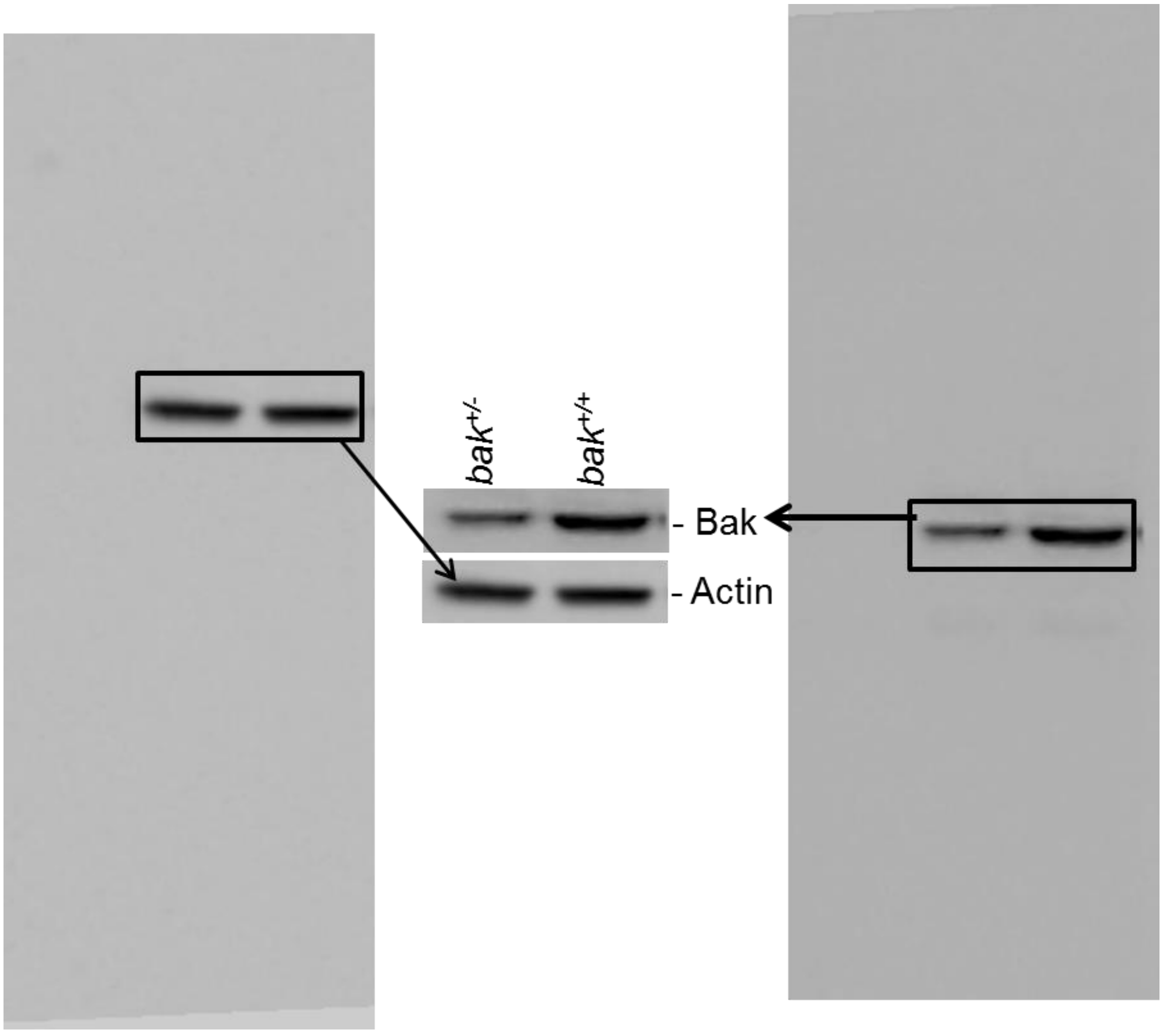

Figure 2E

Supplementary Figure 8. Uncropped images related to Figure 3

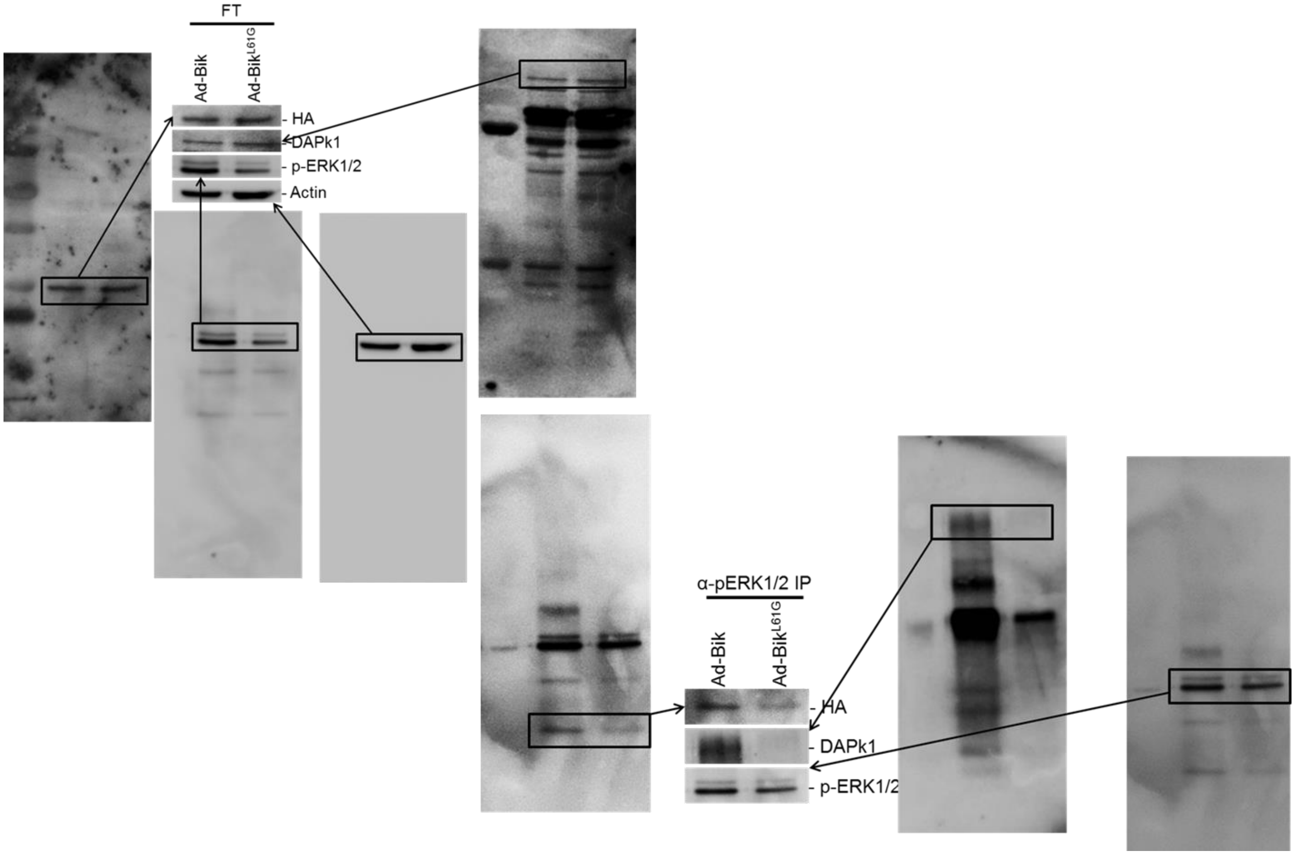

Figure 3A

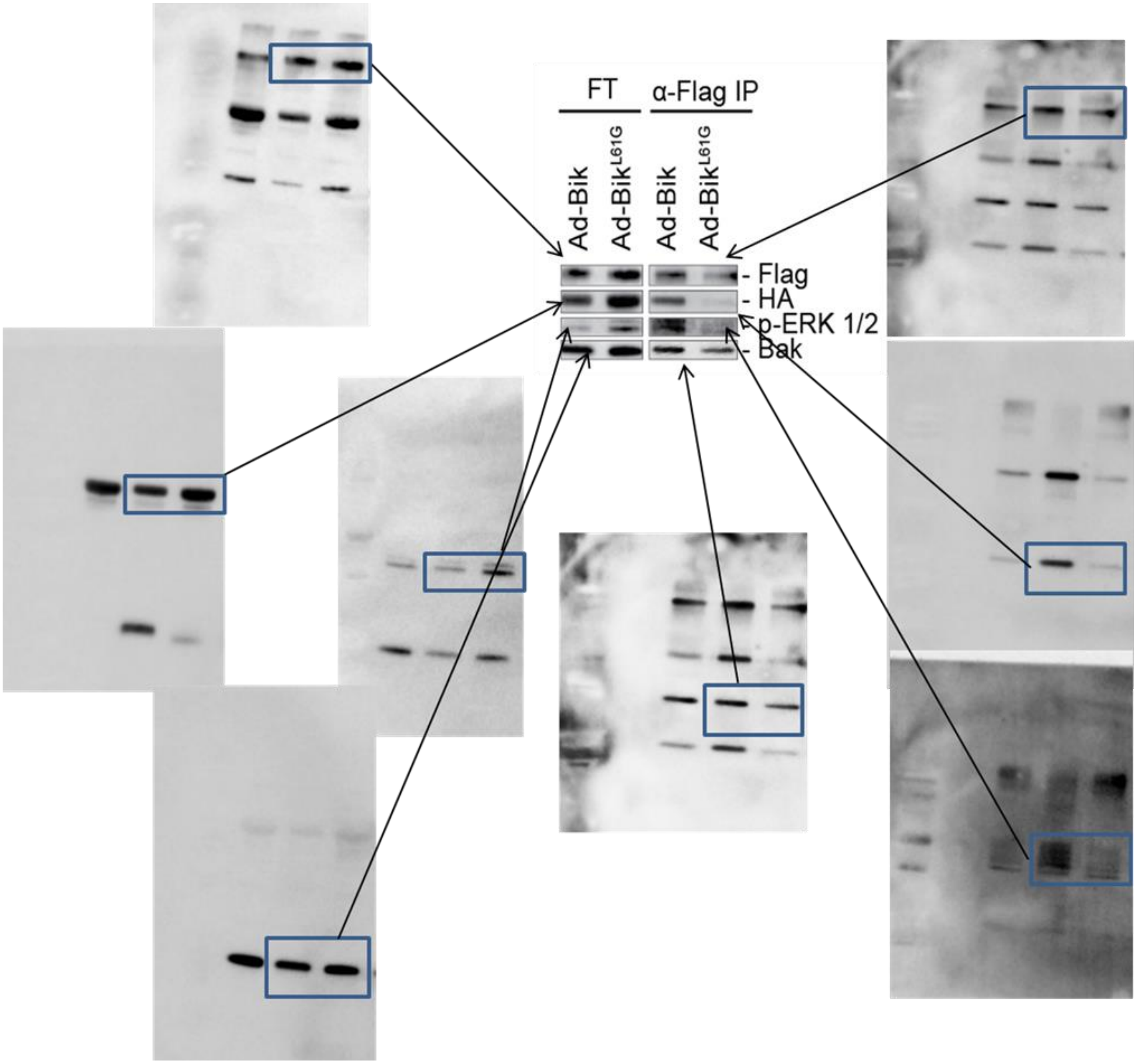
FT
α-Flag IP
Ad-Bik
Ad-Bik^{L61G}
Ad-Bik
Ad-Bik^{L61G}
- Flag
- HA
p-ERK 1/2
- Bak

Figure 3B

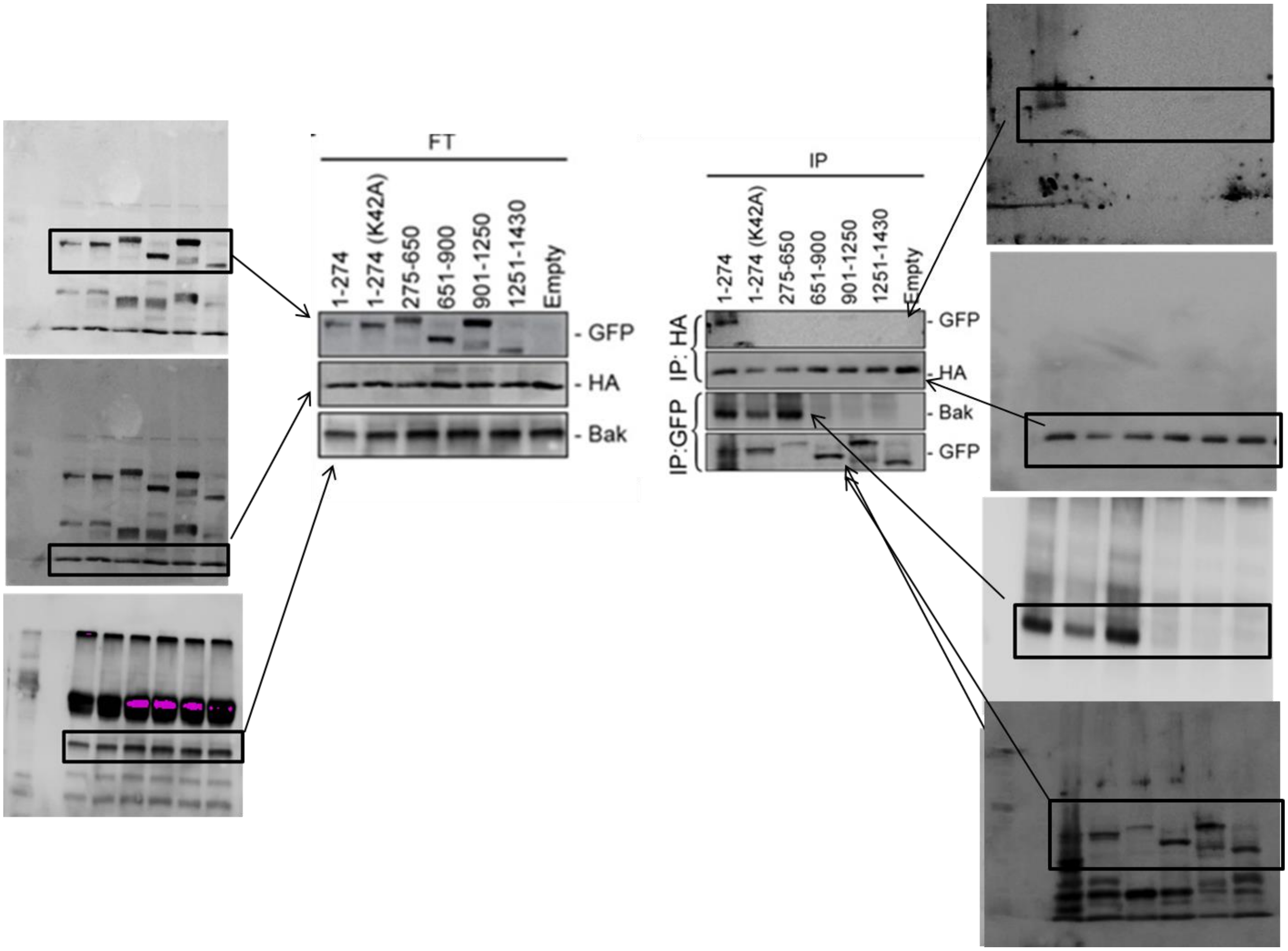
FT
1-274
1-274 (K42A)
275-650
651-900
901-1250
1251-1430
Empty
- GFP
- HA
- Bak
IP
IP: HA
IP:GFP
- GFP
- HA
- Bak
- GFP

Figure 3C

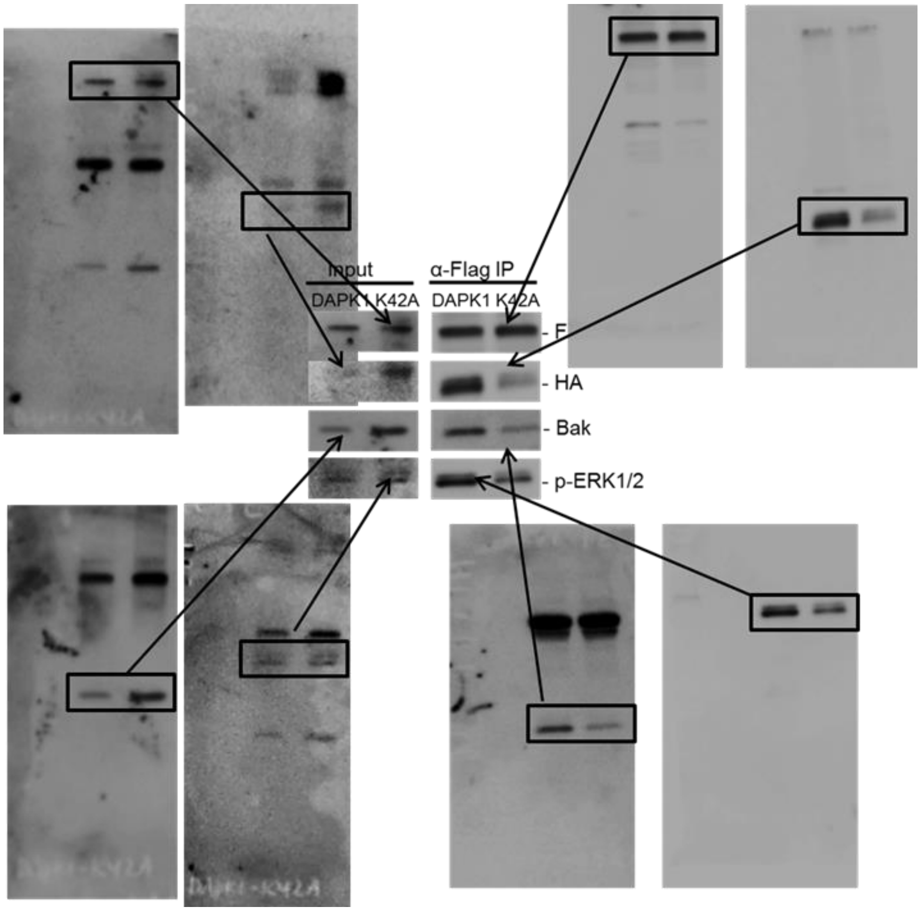

Figure 3D

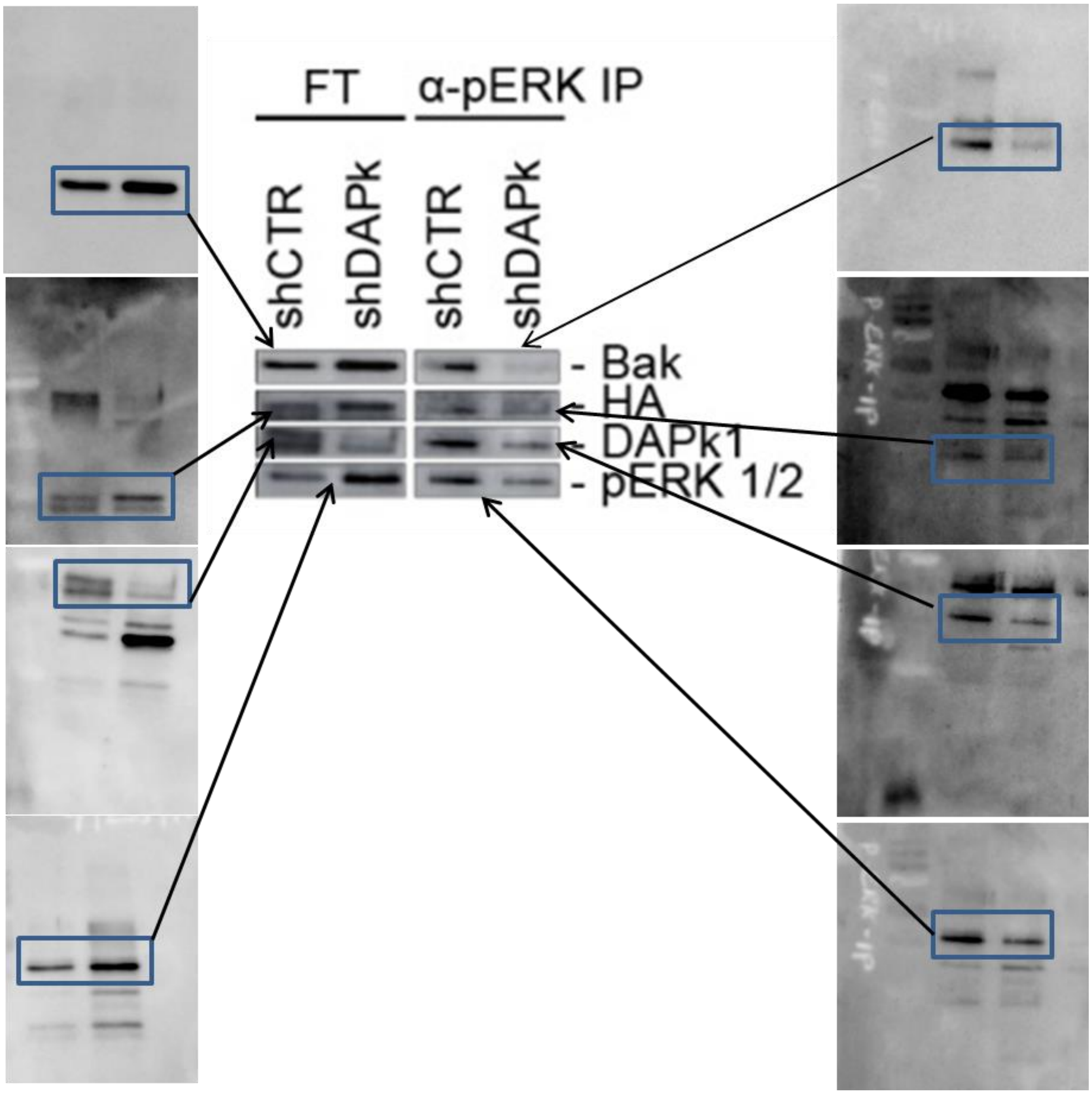

Figure 3F

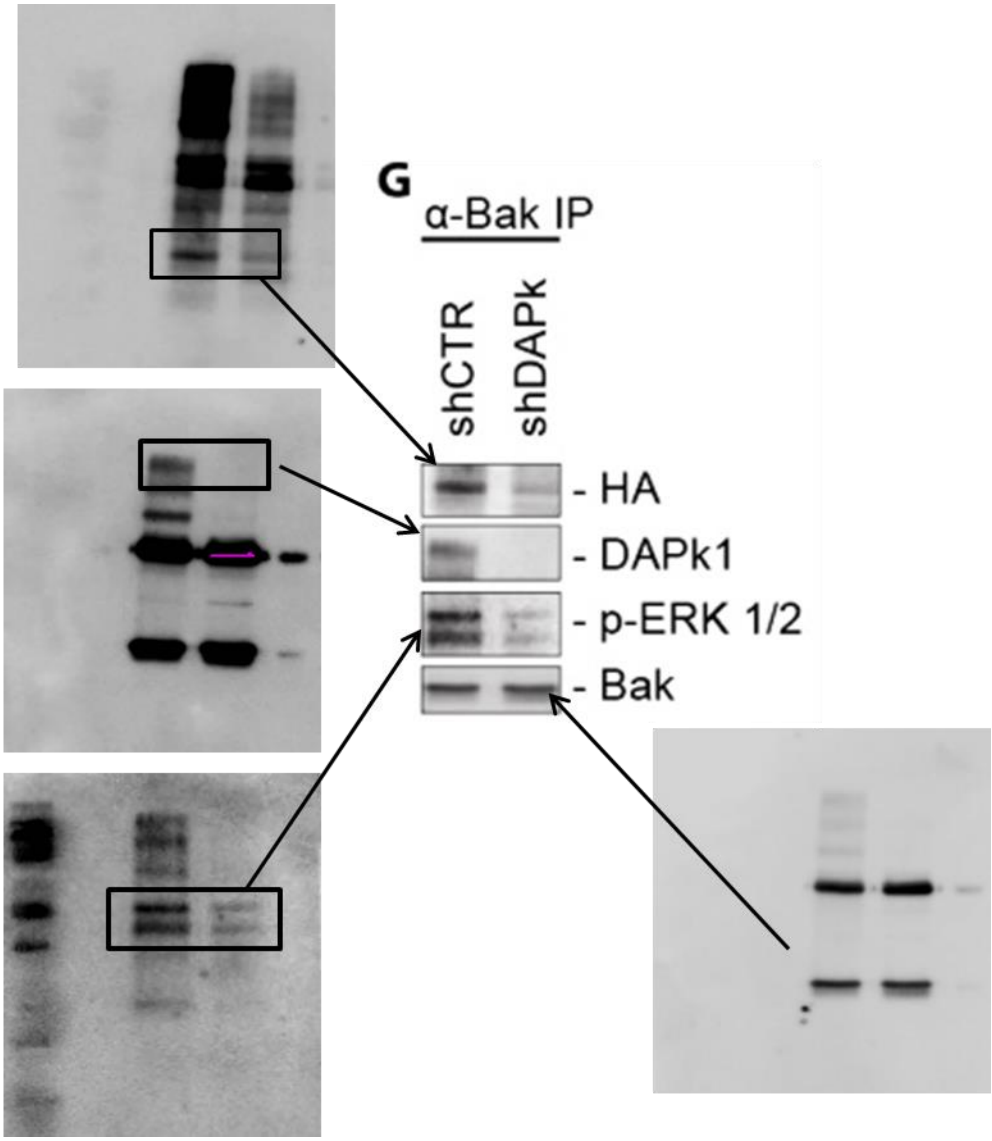

Figure 3G

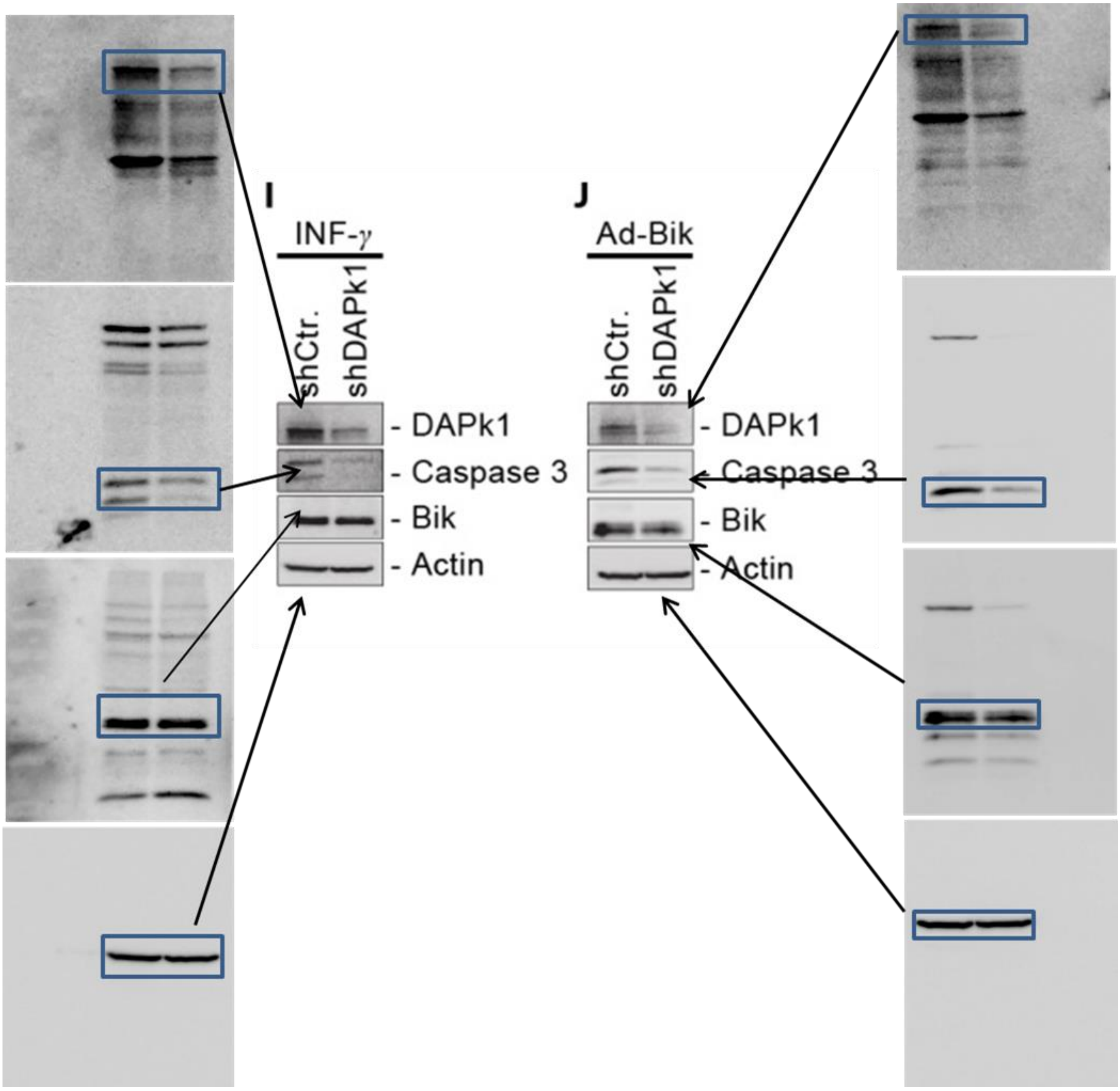

Figure 3I

Figure 3J

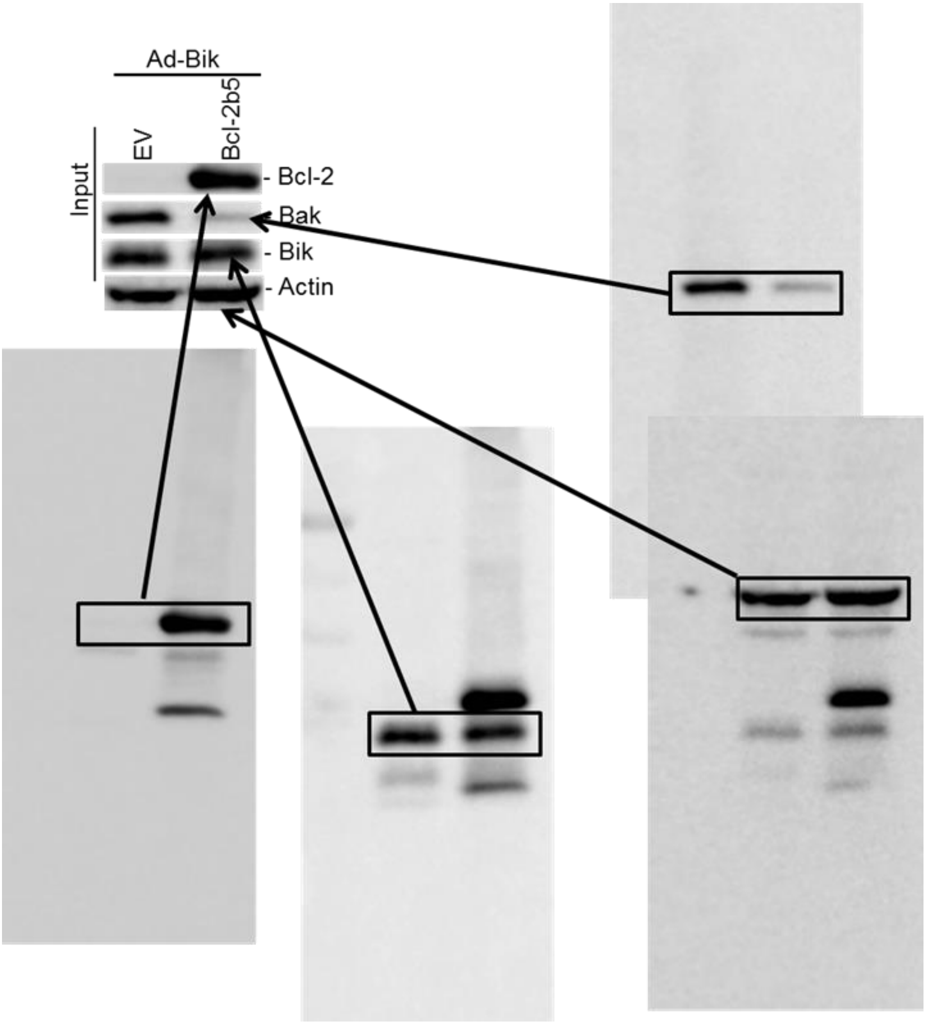

Figure 3L

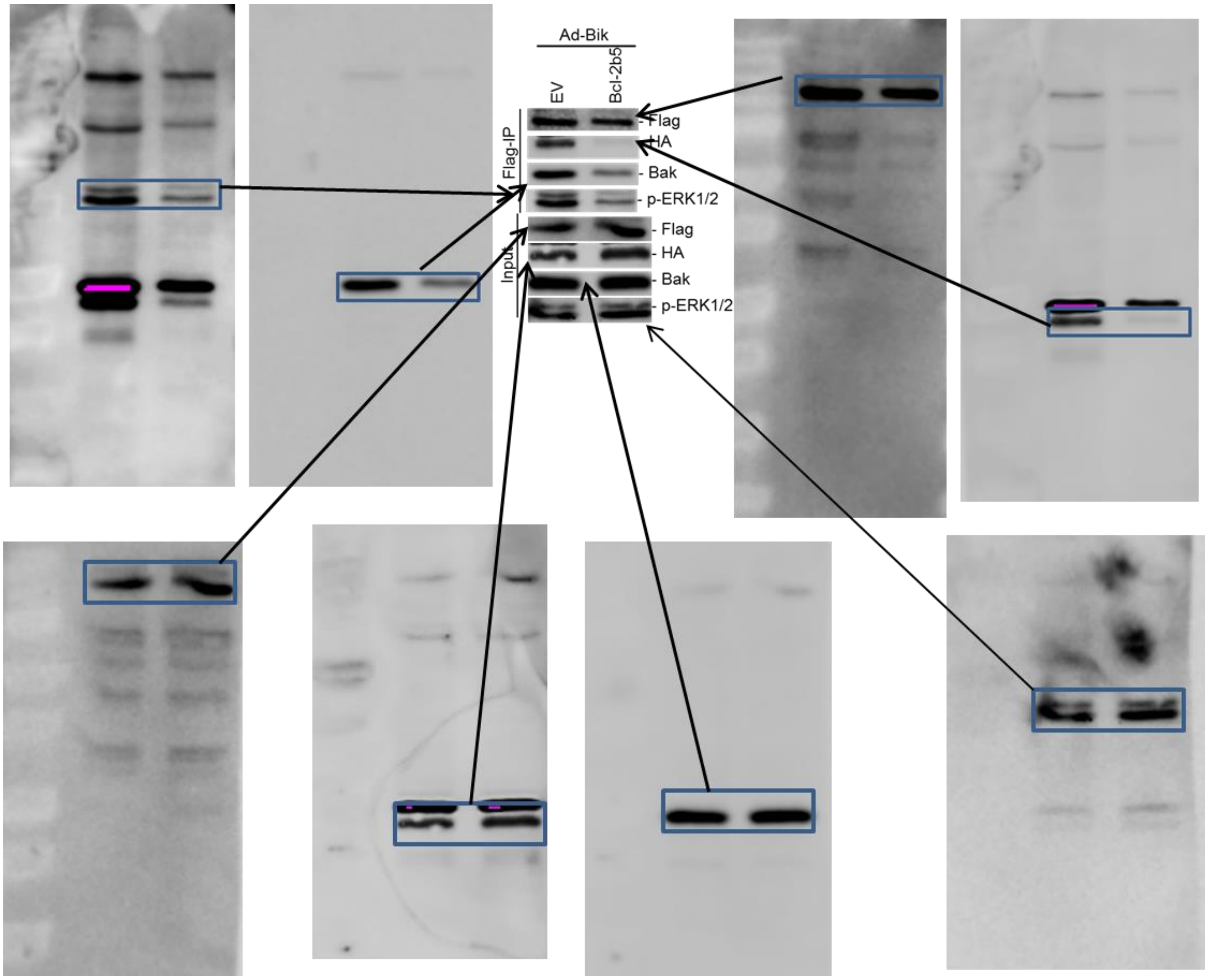
Ad-Bik
EV
Bcl-2b5
Flag-IP
Input
- Flag
- HA
- Bak
- p-ERK1/2
- Flag
- HA
- Bak
- p-ERK1/2

Figure 3M

Supplementary Figure 9. Uncropped images related to Figure 6

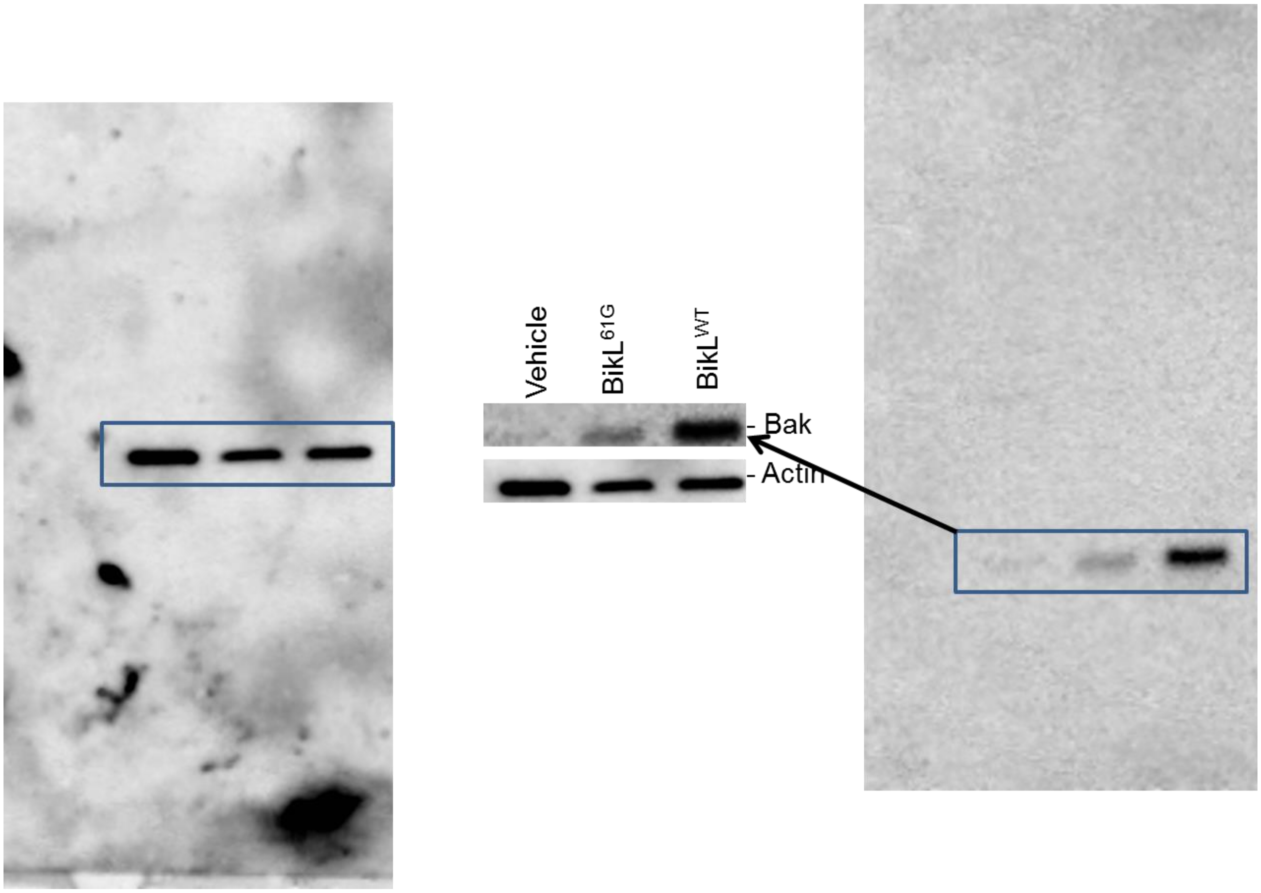

Figure 6B

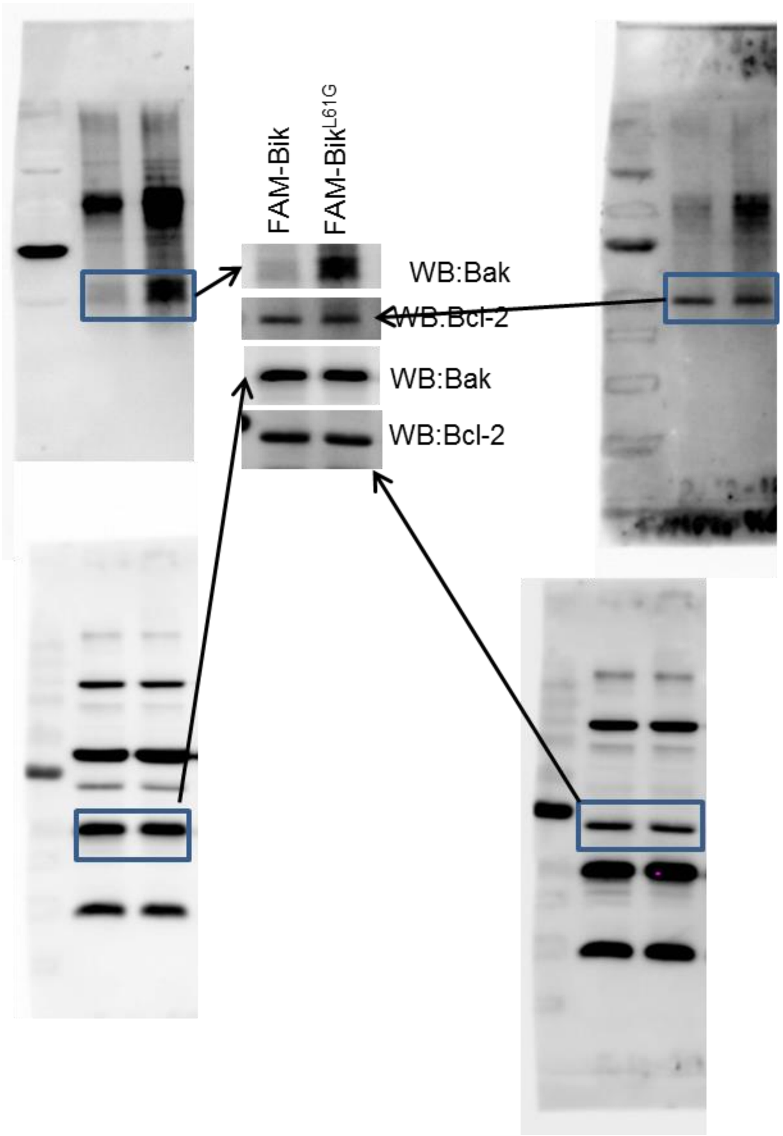

Figure 6C

Supplementary Figure 10. Uncropped images related to Supplementary Figure 1

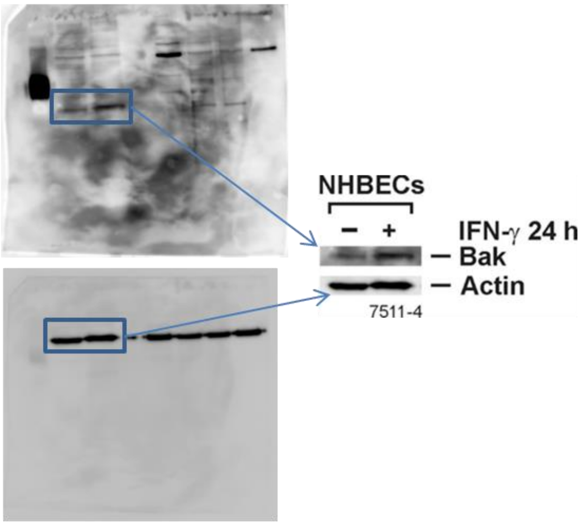

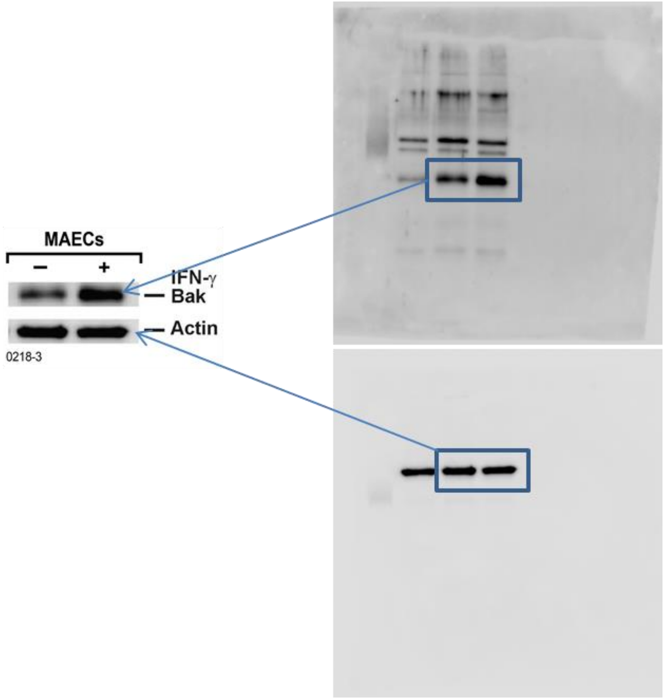

Supplementary Figure 1a

Supplementary Figure 1b

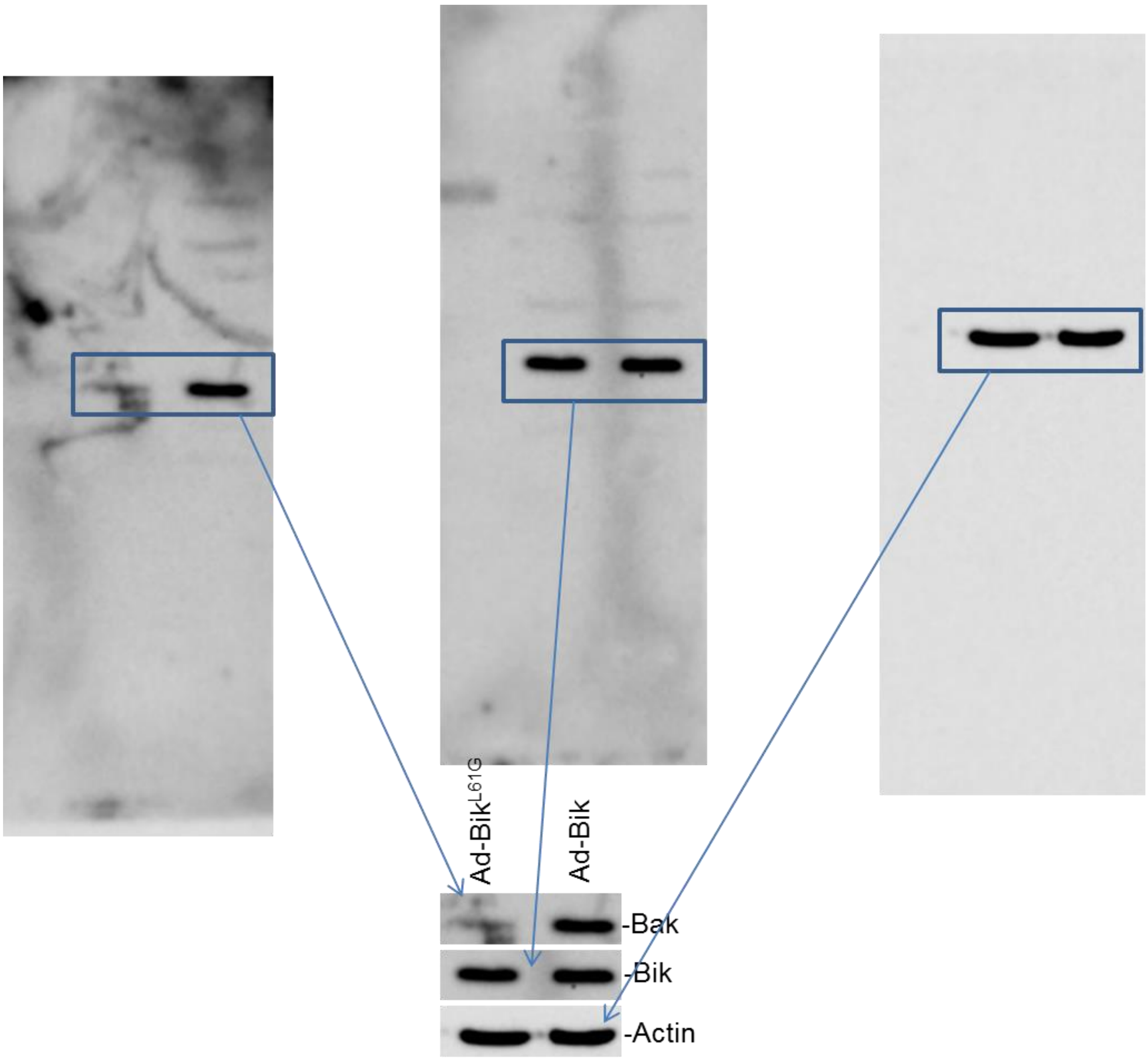

Supplementary Figure 1c

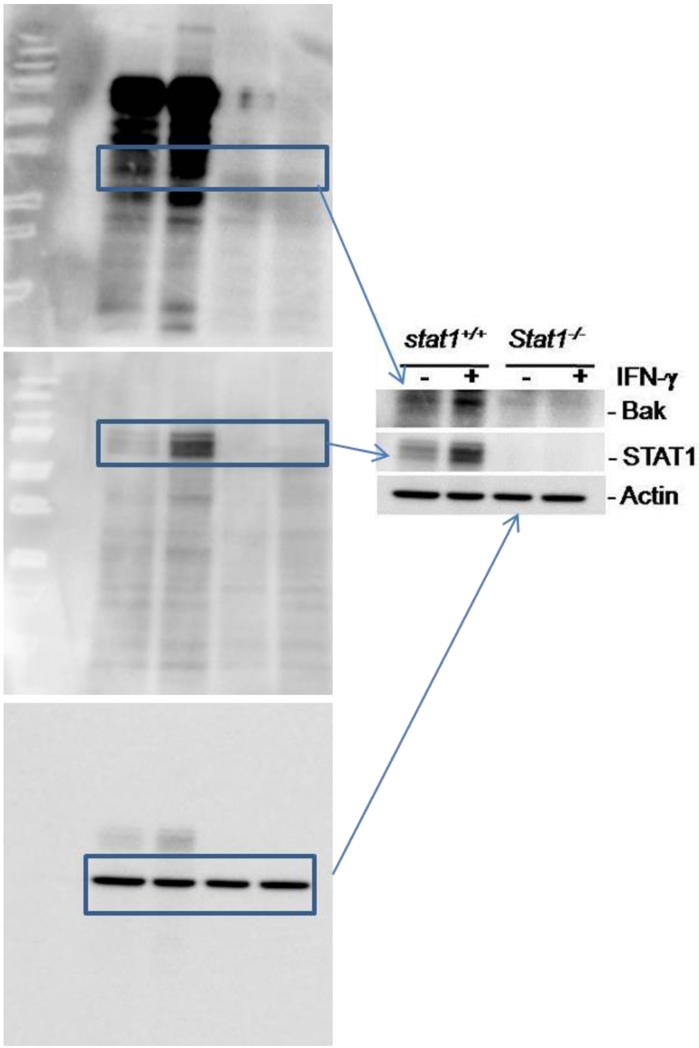

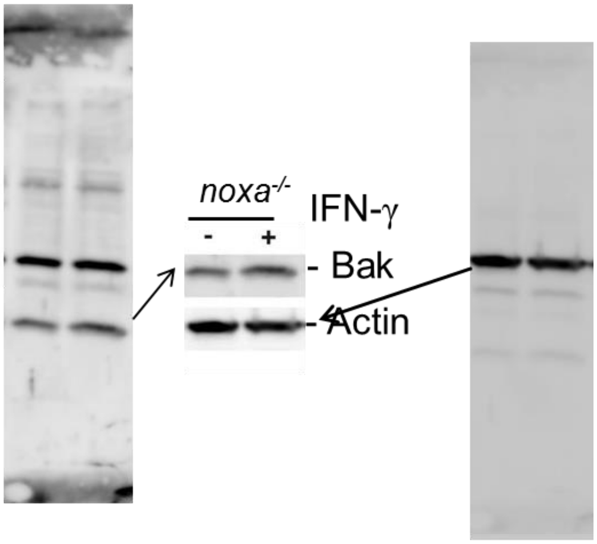

Supplementary Figure 1e

Supplementary Figure 1f

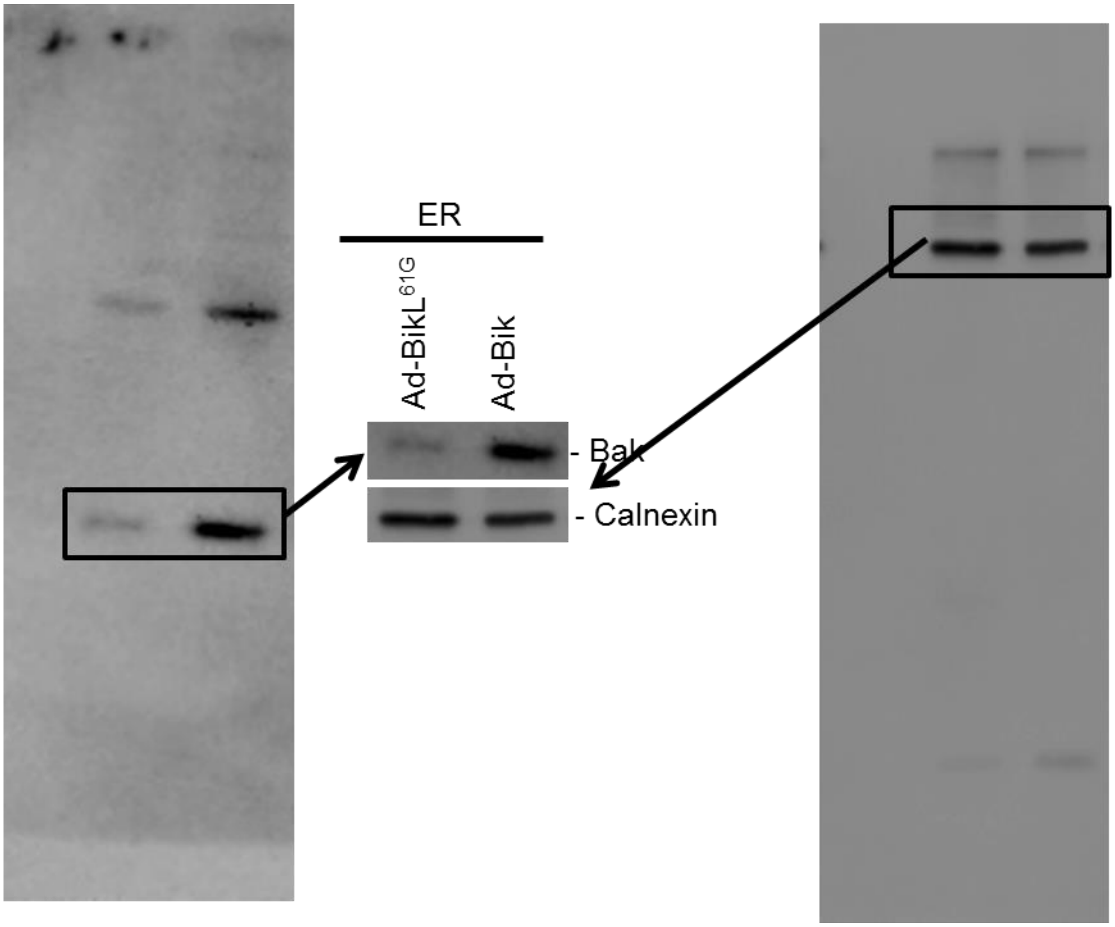

Supplementary Figure 1j

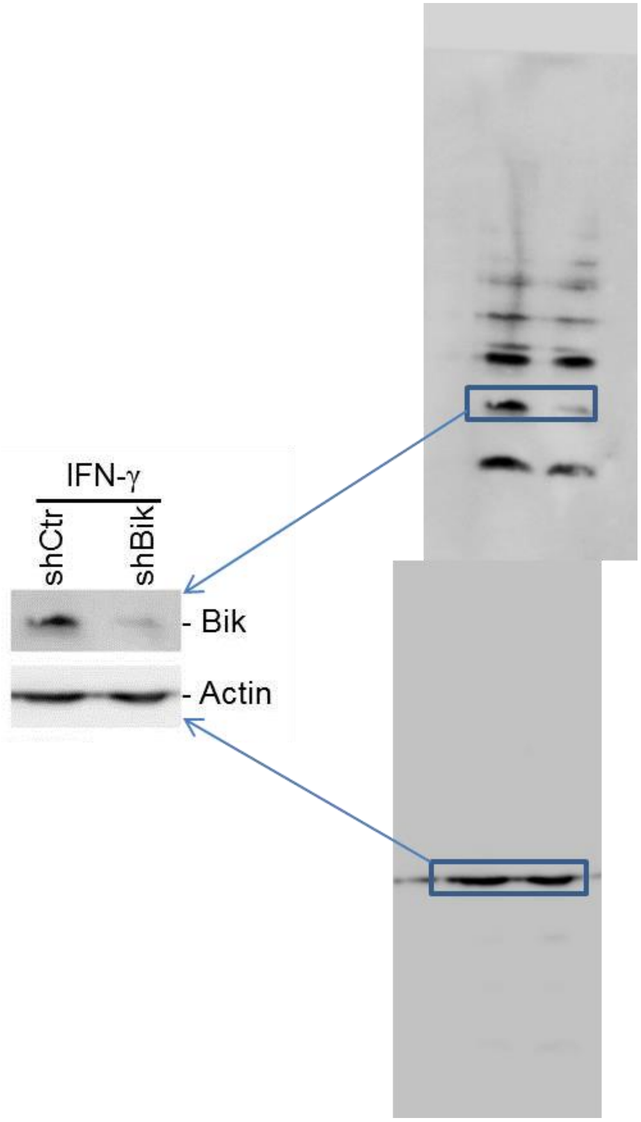

Supplementary Figure 1m

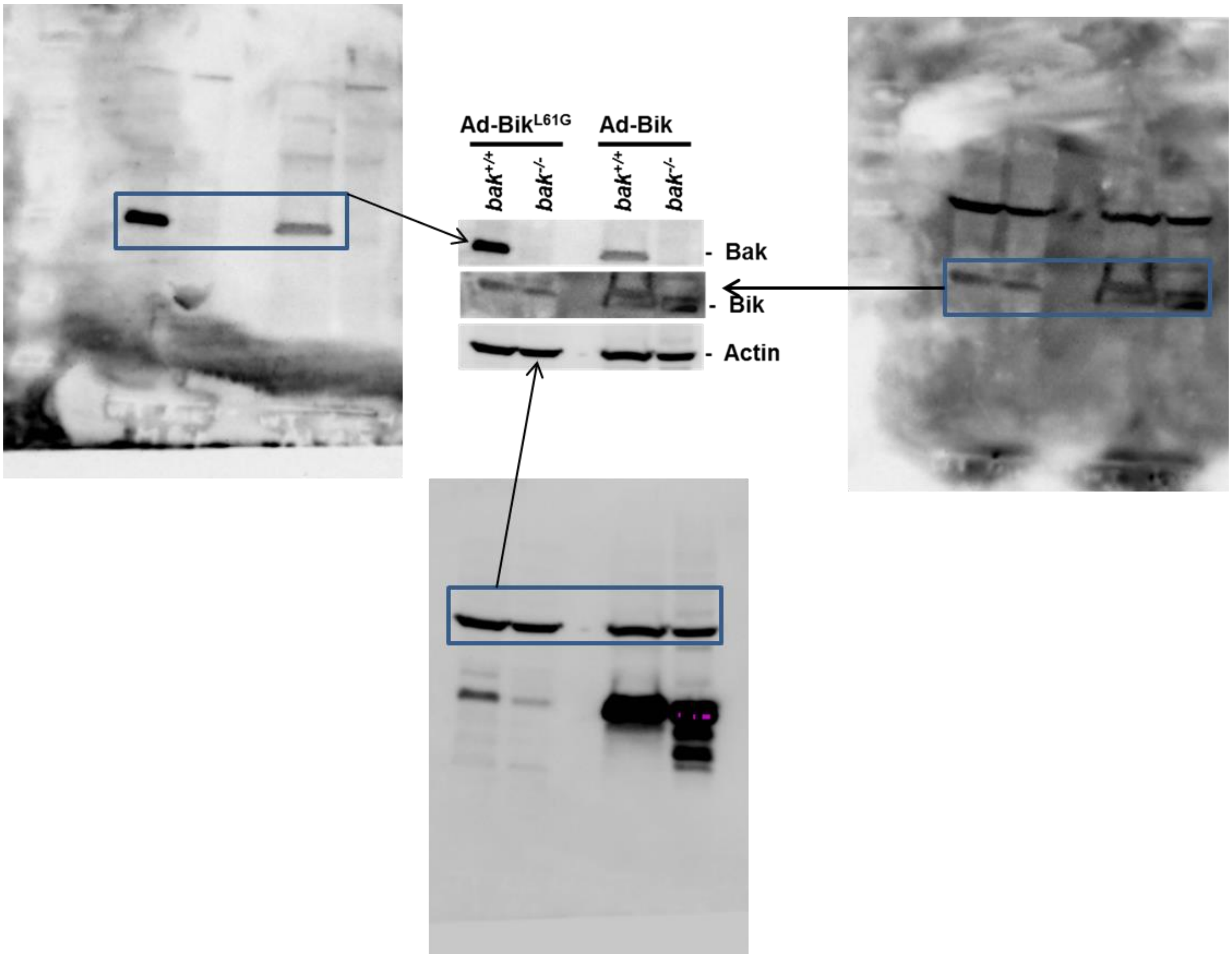

Supplementary Figure 1o

Supplementary Figure 11: Uncropped images related to Supplementary Figure 3

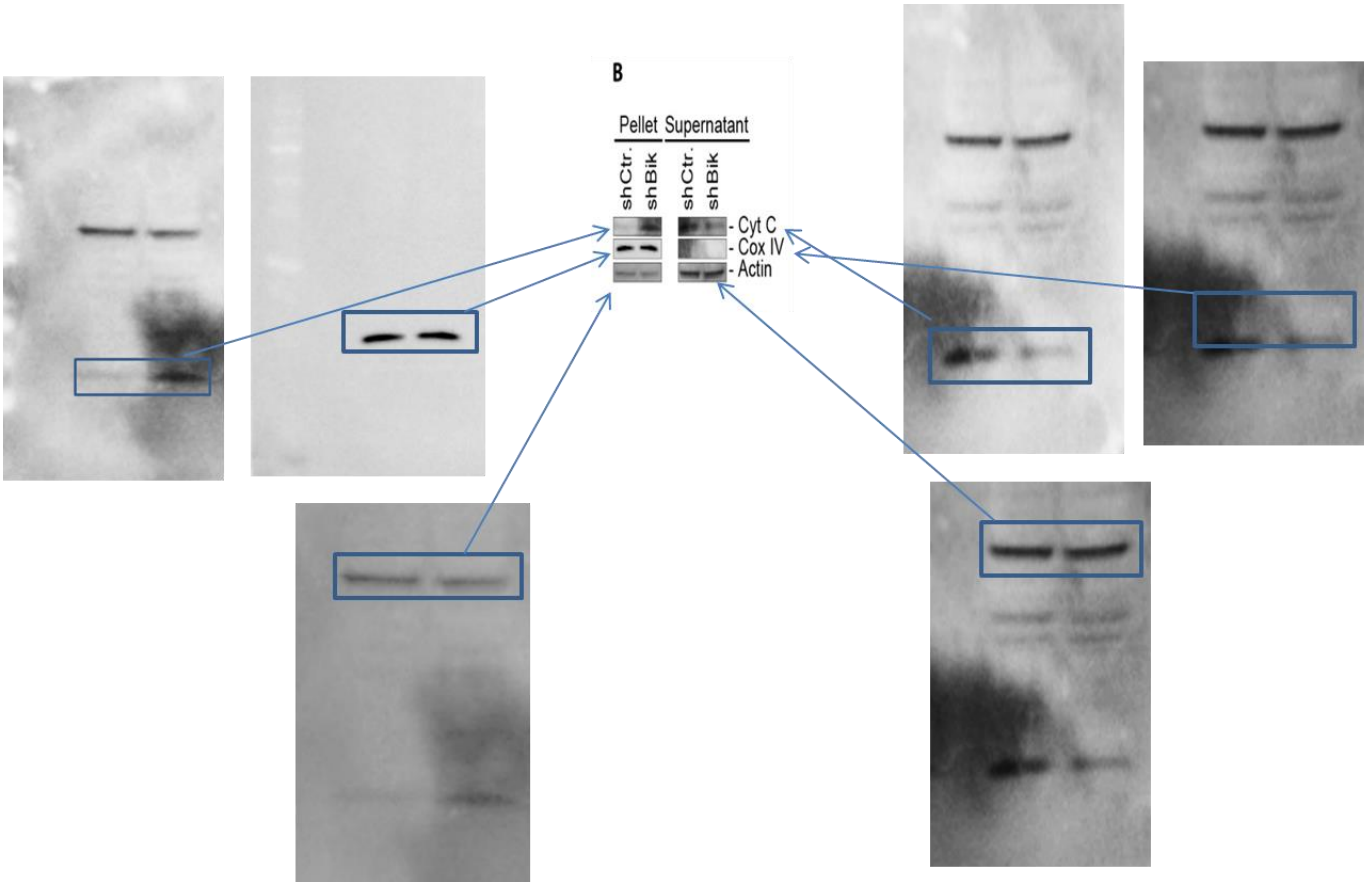

Supplementary Figure 3b

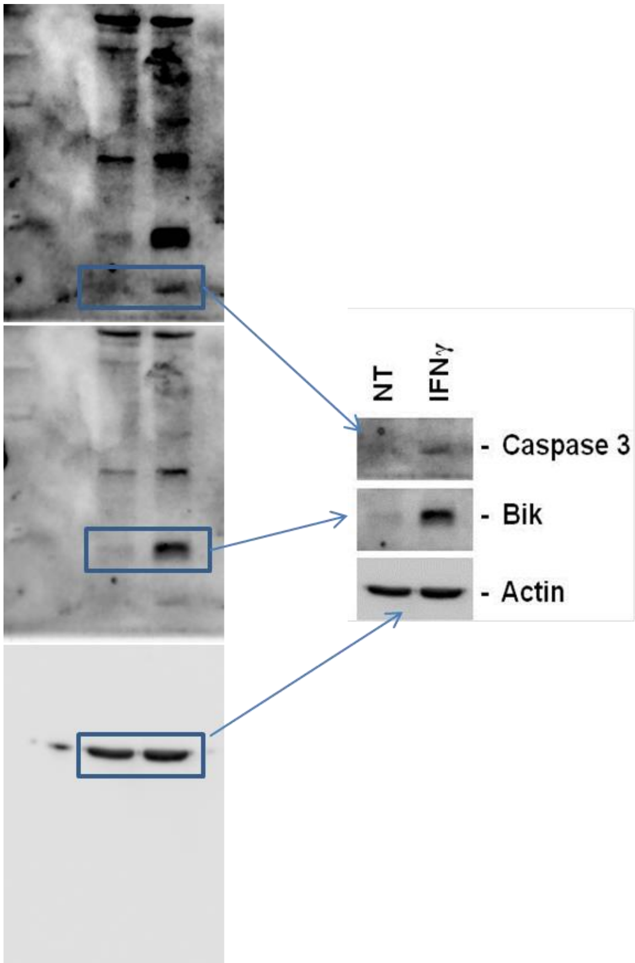

Supplementary Figure 3c

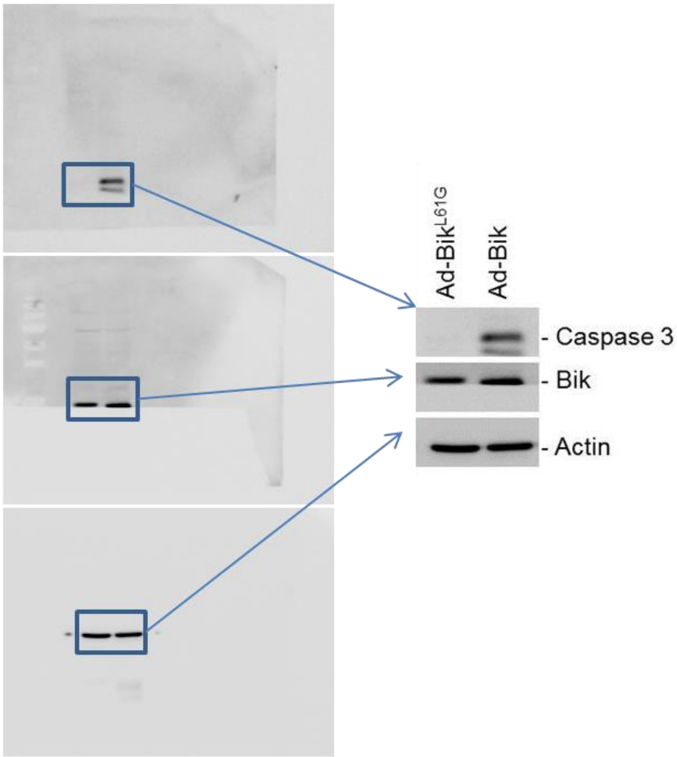

Supplementary Figure 3d

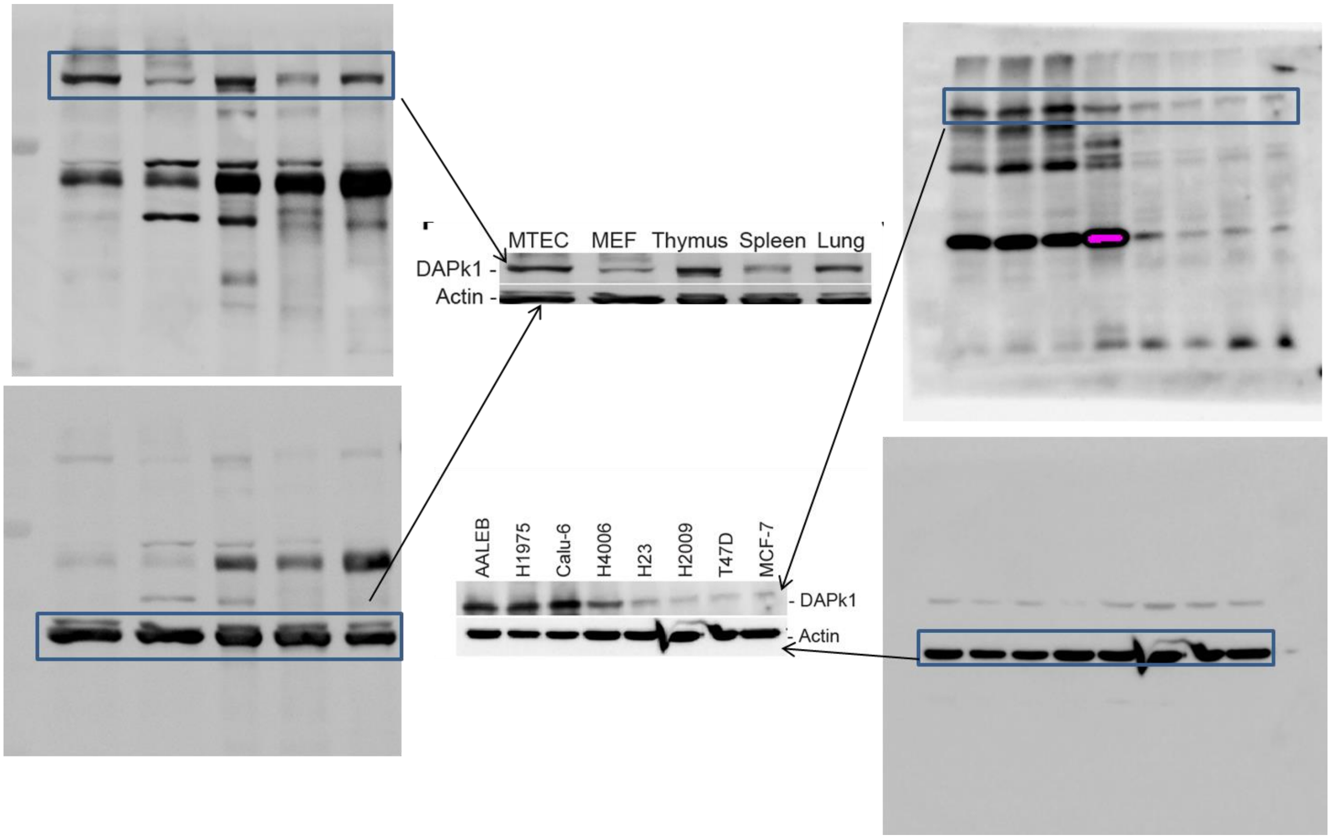

Supplementary Figure 3e **Supplementary Figure 3f**

Supplementary Figure 12

SHS-Bik1^{WT}

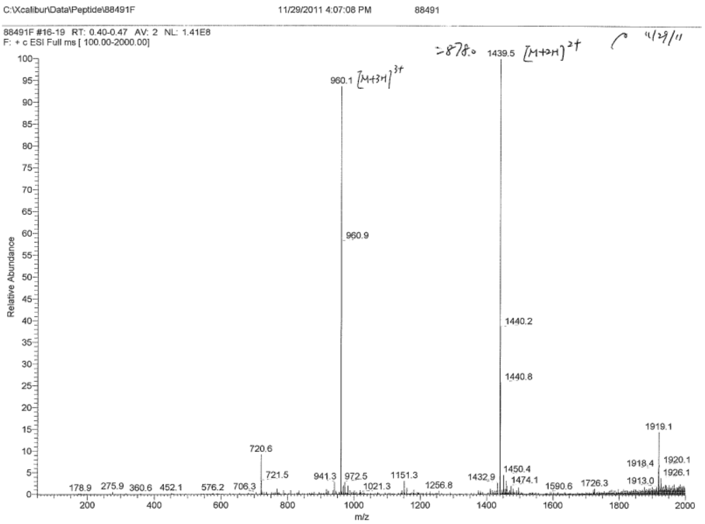

Data File C:\HPCHEM\1\DATA\113011\024-0601.D Page 1 of 1

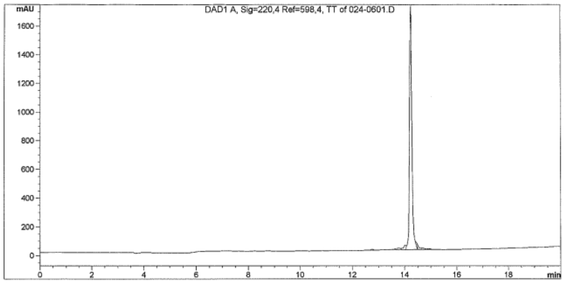

Area Percent Report

Sorted by Retention Time
Analysis Method : C:\HPCHEM\1\METHODS\0-100-20.M
Multiplier : 1.000

Signal 1: DAD1 A, Sig=220,4 Ref=598,4, TT Sample Name: 88491
of: C:\HPCHEM\1\DATA\113011\024-0601.D
at: Wed Nov 30 11:36:06 2011 by: FINAL QC
Acq. Method: 0-100-20.M
Sequence Line: 6 Vial: 24 Inj: 1

Peak #	Ret Time [min]	Sig	Type	Area [mAU*sec]	Height [mAU]	Area %
1	12.757	1	MM	45.591	7.188	0.3788
2	13.762	1	MV	152.657	10.891	1.2685
3	14.099	1	VF	241.930	39.824	2.0103
4	14.240	1	MF R	11249.118	26.789	93.4753
5	14.483	1	MM T	123.812	34.006	1.0288
6	14.644	1	FM	126.506	13.908	1.0512
7	14.913	1	FM	94.703	7.419	0.7869
Totals :				12034.317	140.024	

*** End of Report ***

HP 1090 Win system Wed Nov 30 12:05:55 2011 by FINAL QC

Supplementary Figure 12: High-performance liquid chromatography (HPLC) and Mass spectrometry (MS) data for single hydrocarbon stapled wild-type Bik1 (SHS-Bik1^{WT}) peptide.

Supplementary Figure 13

SHS-Bik2^{WT}

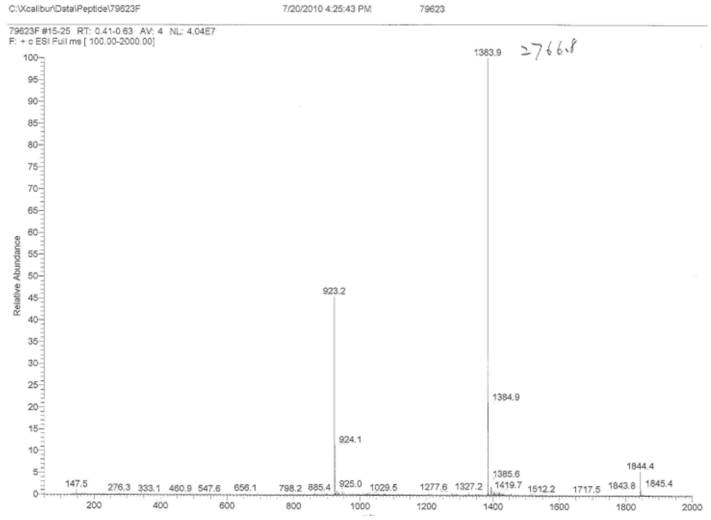

Data File C:\HPCHEM\1\DATA\07212010\023-0401.D Page 1 of 1

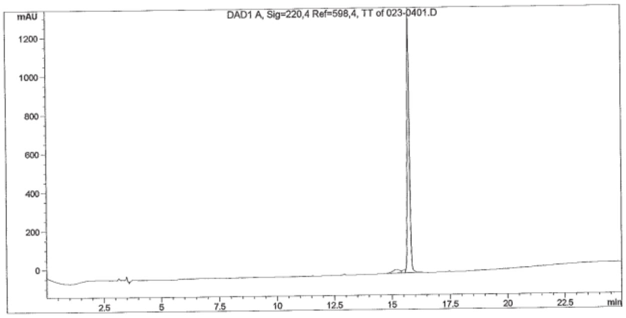


```
==================================================================
                       Area Percent Report
==================================================================

Sorted by Retention Time
Analysis Method    : C:\HPCHEM\1\METHODS\0-100-20.M
Multiplier         : 1.000

Signal 1: DAD1 A, Sig=220,4 Ref=598,4, TT   Sample Name: 79623
      of: C:\HPCHEM\1\DATA\07212010\023-0401.D
      at: Wed Jul 21 08:58:59 2010  by: FINAL QC
      Acq. Method: 0-100-20.M
      Sequence Line: 4   Vial: 23   Inj: 1
```

Peak #	Ret Time [min]	Sig	Type	Area [mAU*sec]	Height [mAU]	Area %
1	15.142	1	MF	449.041	19.825	4.6482
2	15.568	1	MM T	9.678	14.659	0.1002
3	15.739	1	FM R	9201.877	11.927	95.2516
Totals :				9660.597	46.412	

```
==================================================================
                       *** End of Report ***
```

Supplementary Figures 13: High-performance liquid chromatography (HPLC) and Mass spectrometry (MS) data for single hydrocarbon stapled wild-type Bik1 (SHS-Bik2^{WT}) peptide.

Supplementary Figure 14

Scrambled Peptide

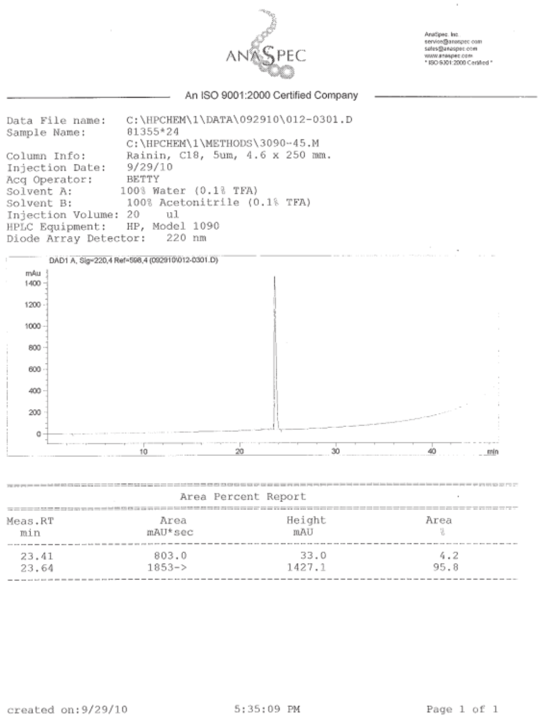

AnaSpec, Inc.
service@anaspec.com
sales@anaspec.com
www.anaspec.com
" ISO 9001:2000 Certified "

An ISO 9001:2000 Certified Company

```
Data File name:   C:\HPCHEM\1\DATA\092910\012-0301.D
Sample Name:      81355*24
                  C:\HPCHEM\1\METHODS\3090-45.M
Column Info:      Rainin, C18, 5um, 4.6 x 250 mm.
Injection Date:   9/29/10
Acq Operator:     BETTY
Solvent A:       100% Water (0.1% TFA)
Solvent B:        100% Acetonitrile (0.1% TFA)
Injection Volume: 20    ul
HPLC Equipment:   HP, Model 1090
Diode Array Detector:   220 nm
```


Area Percent Report

Meas.RT min	Area mAU*sec	Height mAU	Area %
23.41	803.0	33.0	4.2
23.64	1853->	1427.1	95.8

created on:9/29/10   5:35:09 PM   Page 1 of 1

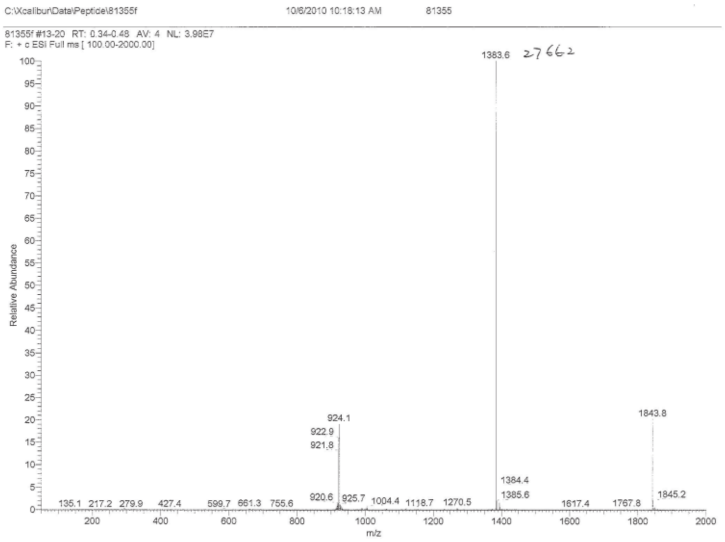

Supplementary Figures 14: High-performance liquid chromatography (HPLC) and Mass spectrometry (MS) data for single hydrocarbon stapled scramble peptide.

Supplementary Figure 15

DHS-Bik1^{WT}

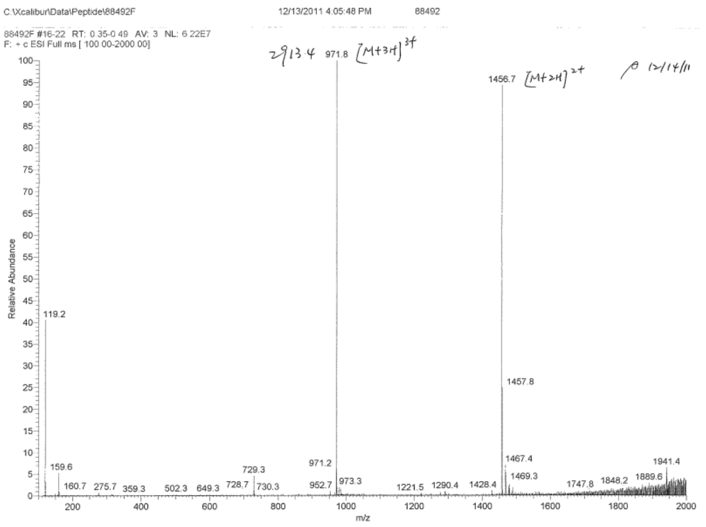

Data File C:\HPCHEM\1\DATA\121311\023-0401.D Sample Name: 88492

```
=====================================================================
Injection Date  : 12/13/11 2:46:30 PM            Seq. Line :   4
Sample Name     : 88492                               Vial :  23
Acq. Operator   : final QC                             Inj :   1
                                                Inj Volume : 5 µl
Different Inj Volume from Sequence !     Actual Inj Volume : 6 µl
Acq. Method     : C:\HPCHEM\1\METHODS\0-100-20.M
Last changed    : 12/13/11 2:45:56 PM by final QC
                  (modified after loading)
Analysis Method : C:\HPCHEM\1\METHODS\0-100-20.M
Last changed    : 8/26/11 8:37:31 AM by final QC
```

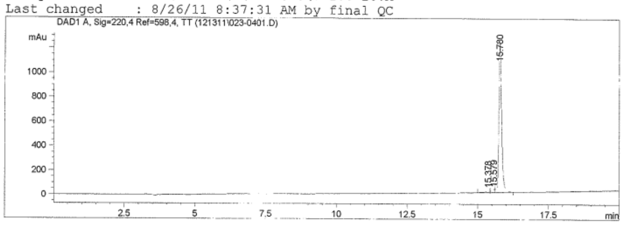


```
=====================================================================
                     Area Percent Report
=====================================================================

Sorted By             :      Signal
Multiplier            :      1.0000
Dilution              :      1.0000

Signal 1: DAD1 A, Sig=220,4 Ref=598,4, TT
 Results obtained with enhanced integrator!

Peak RetTime Type  Width     Area      Height     Area
  #   [min]        [min]   [mAu*s]     [mAu]        %
----|-------|----|-------|----------|----------|--------|
   1  15.378 BV    0.1178  189.11629   21.67778   2.1832
   2  15.579 VV    0.1018  206.02092   27.00637   2.3784
   3  15.780 VB    0.1008 8267.07422 1229.60352  95.4384

Totals :                  8662.21143 1278.28767

=====================================================================
                       *** End of Report ***
```

Supplementary Figures 15: High-performance liquid chromatography (HPLC) and Mass spectrometry (MS) data for single hydrocarbon stapled wild-type Bik1 (DHS-Bik1^{WT}) peptide.

Supplementary Figure 16

DHS-Bik2^{L61G}

Data File C:\HPCHEM\1\DATA\050712\026-0601.D Page 1 of 1

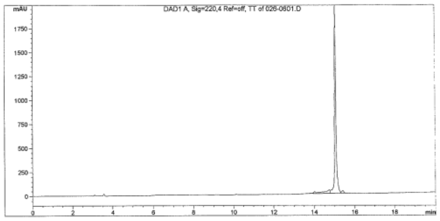

Area Percent Report

Sorted by Retention Time
Analysis Method : C:\HPCHEM\1\METHODS\0-60-20.M
Multiplier : 1.000

Signal 1: DAD1 A, Sig=220,4 Ref=off, TT Sample Name: 90412
of: C:\HPCHEM\1\DATA\050712\026-0601.D
at: Mon May 07 12:23:54 2012 by: FINAL QC
Acq. Method: 0-100-20.M
Sequence Line: 6 Vial: 26 Inj: 1

Peak #	Ret Time [min]	Sig	Type	Area [mAU*sec]	Height [mAU]	Area %
1	14.014	1	FM	183.944	21.375	1.5913
2	14.733	1	FF	795.329	37.622	6.8802
3	14.915	1	MM T	10.440	5.392	0.0903
4	15.038	1	MF R	10365.838	32.074	89.6728
5	15.401	1	FB	204.077	28.660	1.7654
Totals :				11559.627	125.122	

*** End of Report ***

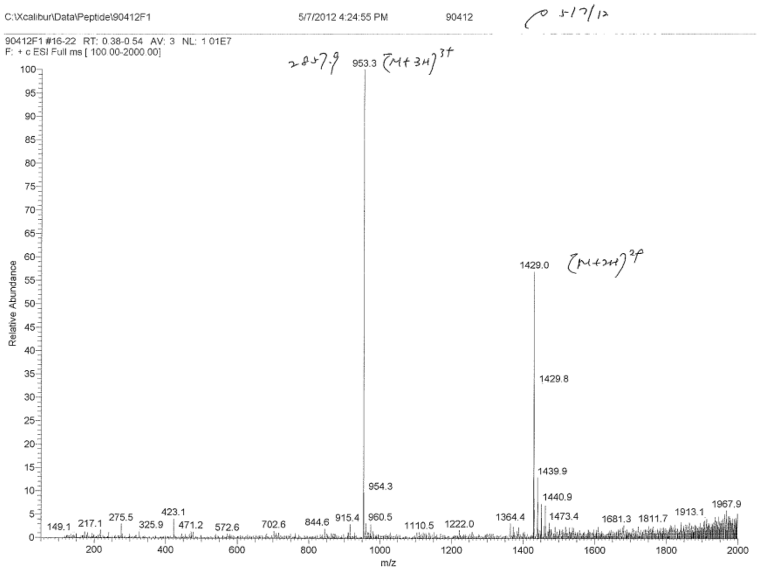

Supplementary Figures 16: High-performance liquid chromatography (HPLC) and Mass spectrometry (MS) data for single hydrocarbon stapled L61G mutant Bik2 (DHS-Bik2^{WT}) peptide.
